# Supplementary material for: PD-L1 recruits phospholipase C and enhances tumorigenicity of lung tumors harboring mutant forms of EGFR
Source: Cell Rep. 2021 May 25;35(8):109181. doi: 10.1016/j.celrep.2021.109181 (PMC8170369; doi:10.1016/j.celrep.2021.109181)
Supplement: Document S1. Figures S1–S7 [file mmc1.pdf]

Cell Reports, Volume *35*

Supplemental information

PD-L1 recruits phospholipase C and enhances tumorigenicity of lung tumors harboring mutant forms of EGFR

Soma Ghosh, Nishanth Belugali Nataraj, Ashish Noronha, Sushant Patkar, Arunachalam Sekar, Saptaparna Mukherjee, Sabina Winograd-Katz, Lior Kramarski, Aakanksha Verma, Moshit Lindzen, Diana Drago Garcia, Joseph Green, Galit Eisenberg, Hava Gil-Henn, Arkaprabha Basu, Yan Lender, Shimon Weiss, Moshe Oren, Michal Lotem, Benjamin Geiger, Eytan Ruppin, and Yosef Yarden

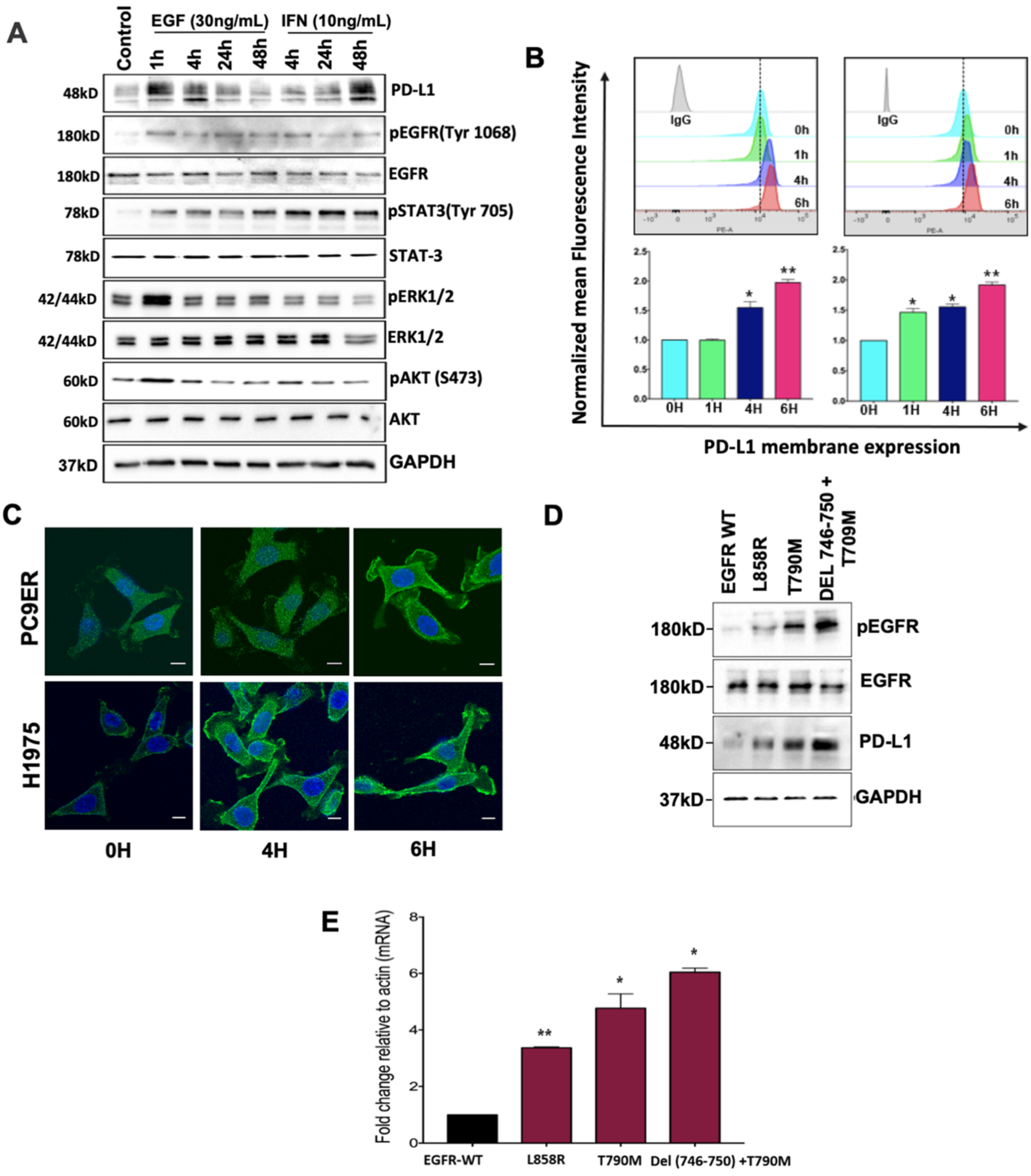

Figure S1: Delayed and early induction of PD-L1 by IFN-g and EGF (respectively), related to Figure 1. (**A**) Serum-starved PC9ER cells were treated for the indicated time intervals with EGF or IFN-

g. Cleared extracts were immunoblotted for the indicated proteins. (**B**) PC9ER (left) and H1975 (right) cells were stimulated with EGF (30 ng/ml) and later analyzed for PD-L1's surface expression using flow cytometry. Normalized surface levels from 3 experiments are shown. (**C**) PC9ER and H1975 cells ($0.5X10^6$) were seeded on coverslips and allowed to grow for 24 hours. Cells were serum starved for 16 hours, treated with EGF (30 ng/ml), as indicated, and then fixed in formaldehyde (4%). Thereafter, cells were incubated overnight with an anti-PD-L1 primary antibody (green), followed by a secondary, FITC-conjugated antibody. DAPI staining (blue) indicates locations of nuclei. Images were captured using a confocal microscope (63X magnification). Bars, 20 μm. Images are representative of two independent experiments. (**D**) NL20 lung epithelial cells were stably transfected with a vector (pLex307) encoding the indicated mutant forms of EGFR (L858R, T790M or the double mutant del (746-750) plus T790M). Cleared whole cell extracts were immunoblotted for the indicated proteins. GAPDH was used as loading control. Data are representative of two independent experiments. (**E**) The indicated cell lines were analyzed using qPCR for mRNA levels corresponding to PD-L1.

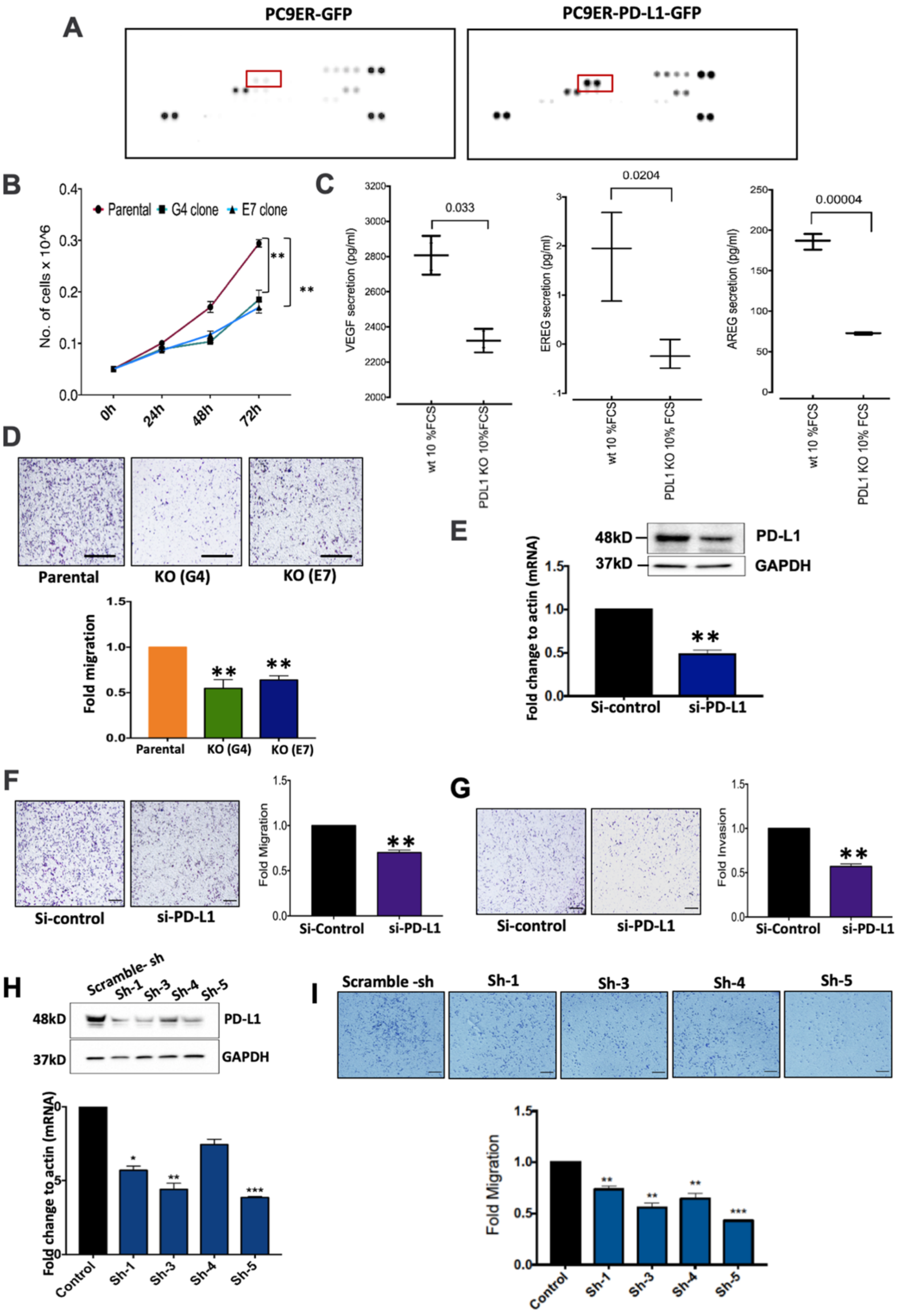

Figure S2: PD-L1 regulates proliferation, migration and invasion of NSCLC cells, as well as the secretome of lung cancer cells, related to Figures 3 and 4. (**A**) Serum-containing media were

conditioned over a period of 72 hours by control PC9ER-GFP cells or cells transiently overexpressing a PD-L1 protein fused to GFP. Cleared media were incubated with cytokine arrays (Proteome Profiler Human Cytokine Array, ARY005B; from R&D Systems). Each array was pre-spotted with 36 different antibodies specific to human cytokines (in duplicates). The arrays were processed according to the manufacturer's instructions. Duplicate spots corresponding to IL-18 are highlighted. (**B**) Parental PC9ER cells (PD-L1-WT; $8X10^4$) and two derivatives PD-L1-KO cells were seeded onto 6-well plates for assessing cell proliferation. Cells were treated with trypsin and counted at the indicated time intervals. Results are presented as means ± S.D. of triplicates. The experiment was repeated thrice. (**C**) Serum-containing media were conditioned over a period of 72 hours by control PC9ER cells or by PD-L1 KO cells. ELISA kits (DuoSet, from R&D Systems), along with internal references, were used to determine concentrations of VEGF, epiregulin and amphiregulin. (**D**) Parental PC9ER cells and the PD-L1-KO clones were seeded in the upper compartment of Transwell migration chambers. Following 18 hours of incubation, four microscope fields were randomly selected for signal quantification. (**E**) PC9ER cells were transfected with siRNA oligonucleotides specific to PD-L1, or with control oligonucleotides (5 nM). Forty-eight hours later, cells were harvested in duplicates for immunoblotting and qPCR analysis. (**F** and **G**) PC9ER cells were treated with siRNAs as in E and 24 hours later they were seeded in the upper compartment of Transwell migration chambers (F) or in Matrigel-coated invasion chambers (G). Thereafter, complete medium was added into the lower compartments and cells were incubated for 18 hours at 37°C. Paraformaldehyde was used to fix cells that reached the lower side of the intervening filter, and crystal violet was used to stain the cells. Four microscope fields were randomly selected for signal quantification. (**H**) PC9ER cells were infected with lentivirus-based shRNAs, which were used to stably knockdown expression of PD-L1. Four different shRNA plasmids were used for transfection into PC9ER cells and four shPD-L1 stable clones were selected. Cells were harvested and extracts were used for either immunoblotting for PD-L1 expression, relative to shCTRL cells (upper panel), or for qPCR tests that assayed the levels of PD-L1's mRNA relative to GAPDH transcripts (lower panel). (**I**) Transwell migration assays were performed with shCTRL-transfected PC9ER cells, along with four different shPD-L1 clones. Bar graphs are presented as means + S.D. of triplicates.

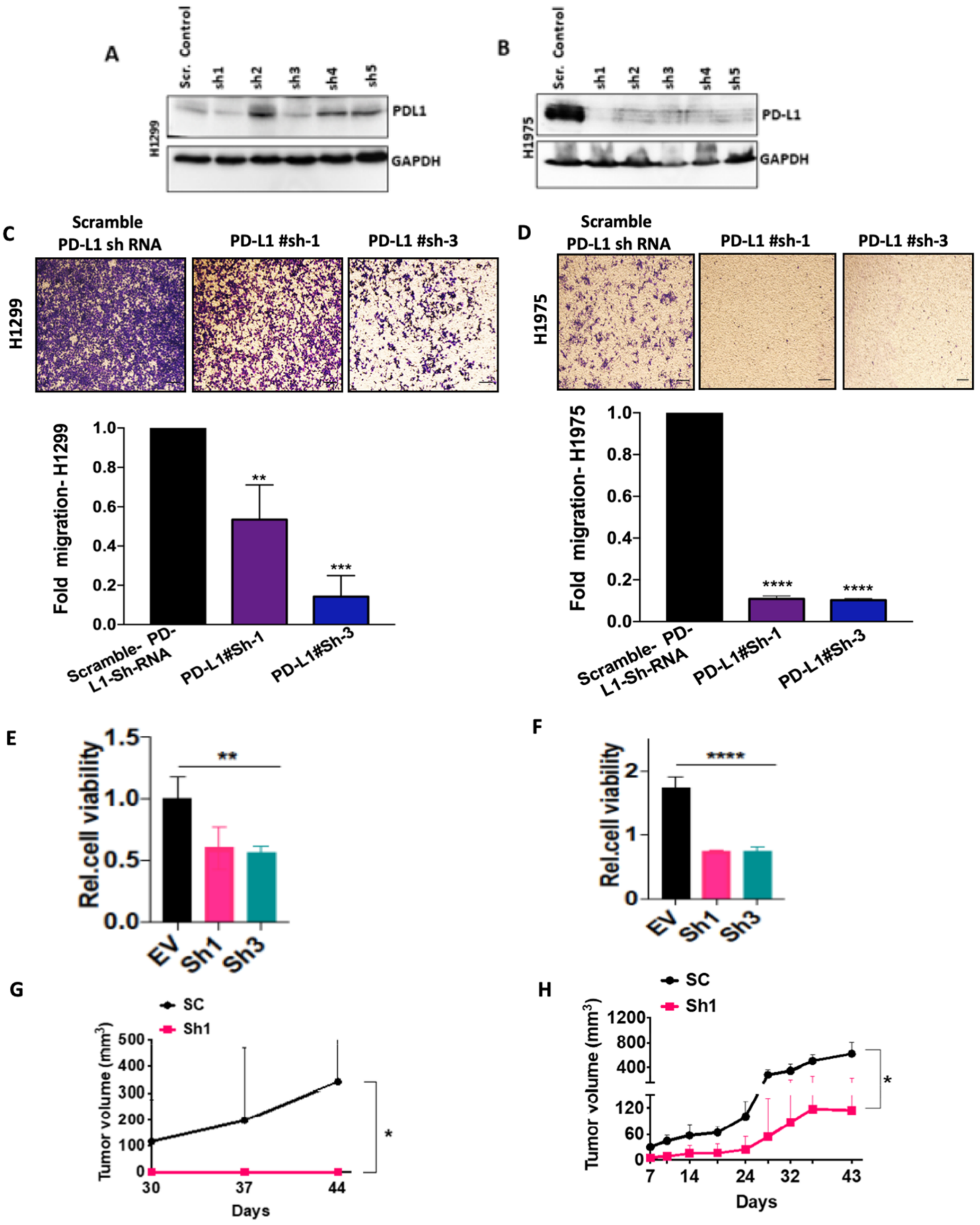

Figure S3: Reduced abundance of PD-L1 results in slower rates of migration, proliferation and tumorigenesis of cells expressing WT-EGFR or the L858R mutant form, related to Figure 4. (**A** and

B) H1299 (WT-EGFR; A) and H1975 (L858R-EGFR; B) cells were infected with lentivirus-based shRNAs, to stably knockdown expression of PD-L1. Five different shRNAs were introduced into each cell line. Cells were harvested and extracts were used for PD-L1 immunoblotting, relative to the Scrambled Control shRNA (SC). GAPDH served as the loading control. (**C** and **D**) Two independent shPD-L1 stable clones (Sh#1 and Sh#3) were selected and Transwell migration assays were performed in H1299 (left panel) and H1975 (right panel), relative to cells treated with the Scrambled Control shRNA (SC). Bar graphs show means + S.D. of triplicates. (**E** and **F**) MTT assays were performed to measure proliferation of H1299 (E) and H1975 cells (F) in which PD-L1 was stably knocked-down. Data presented are means + S.D. of triplicates. (**G** and **H**) shPD-L1 derivatives of H1299 (G) and H1975 cells (H), along with cells treated with Scrambled Control shRNA (SC), were implanted subcutaneously in athymic mice. Shown are averages + S.D. of tumor volumes.

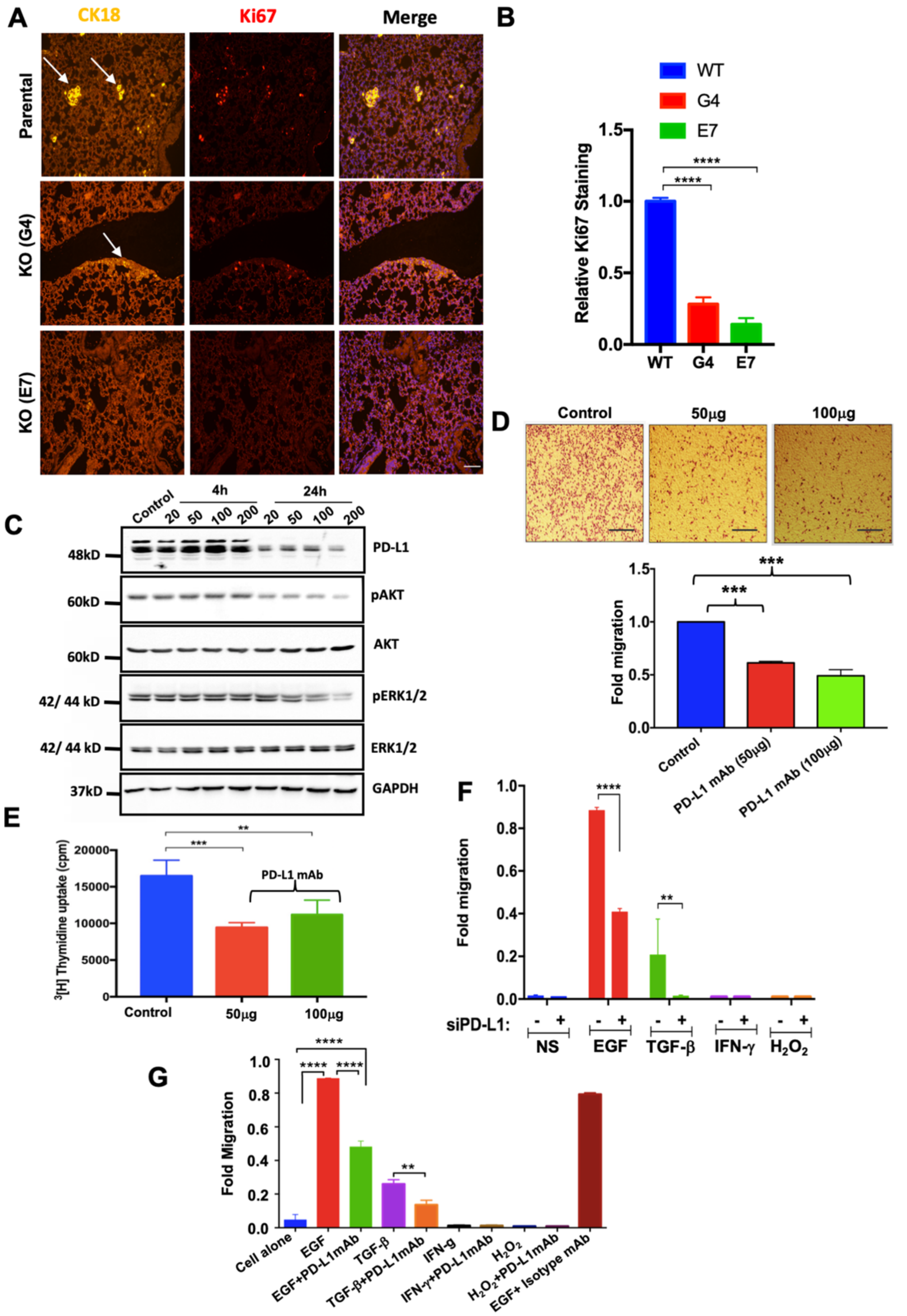

Figure S4: Genetic depletion of PD-L1 retards metastatic colonization of murine lungs, while antibody-induced depletion inhibits growth factor mediated cell proliferation and migration, related to Figure 4. (**A**) Parental PC9ER-LUC (WT), PDL1-KO-G4-LUC and PD-L1-KO-E7-LUC cells

($1X10^6$ cells) were injected into the tail vein of male NSG mice. Ten days post implantation, mice were injected intraperitoneally with 0.05 ml D-Luciferin solution (15 mg/ml, in saline), 5 minutes prior to luminescence measurements. Lungs were excised, fixed in formalin, and paraffin embedded. Sections of 0.2 mm were deparaffinized and immunostained for Ki67. Likewise, we probed thin slices with an antibody specific to the human form of cytokeratin 18. White arrows mark CK18 positive cells. (**B**) Proliferation was quantified by counting Ki67-positive cells in five representative microscope fields per lung (from A). Three lungs were surveyed per group. Scale bars, 0.1 mm. (**C**) PC9ER cells were incubated in the presence of increasing concentrations of durvalumab (20-200 μg/ml). Cells were harvested at the indicated time points. Cleared cell extracts were resolved and immunoblotted as indicated. (**D**) PC9ER cells ($4X10^5$) were pre-treated for 24 hours with durvalumab (0.1 mg/ml or 0.05 mg/ml) and later subjected to a cell migration assay (18 hours). Cell images and average signals are shown. (**E**) PC9ER cells were plated onto 24-well plates at $1X10^4$ cells/well and treated for 48h with durvalumab (0.1 mg/ml or 0.05 mg/ml). Thereafter, the media were refreshed and 3H-thymidine (1 μCi) was added. The incubation was terminated 48 hours later. Shown are averages + S.D. of quadruplicates. (**F**) MCF10A cells were transfected with siPD-L1 or control oligonucleotides, and 36 hours later cells ($4X10^4$) were plated in the upper compartment of 24-well Transwell chambers, in the absence of serum. Thereafter, the serum-free medium in the lower compartment of the chamber was supplemented with growth factors (EGF, 30 ng/ml; TGF-b1, 30 ng/ml; IFN-g, 10 ng/ml) or H2O2, 10 μM). Cells were allowed to migrate for 22 hours at 37°C through the intervening nitrocellulose membrane (8-μm pore size). The filter was later removed, and attached cells were fixed for 15 minutes in saline containing paraformaldehyde (4%). Staining with crystal violet followed this step. Cells growing on the upper side of the filter were removed, while cells located on the bottom side were photographed and optical density was measured at 595 nm. Representative microscope fields were taken, and signals were quantified (means + SEM). (**G**) MCF10A cells ($4X10^5$) were pre-treated for 24 hours with either durvalumab (0.05 mg/ml) or an isotype-matched antibody. Thereafter, the cells were suspended in serum free medium containing the respective antibody and seeded in the upper compartment of Transwell chambers. In parallel, the lower compartment was supplemented with the indicated growth factors/agents (EGF, 30 ng/ml; TGF-b, 30 ng/ml; IFN-g, 10 ng/ml or H2O2, 10 μM) or medium alone. Cells were allowed to migrate for 22 hours. Signal quantification (average + SEM) and representative fields are presented.

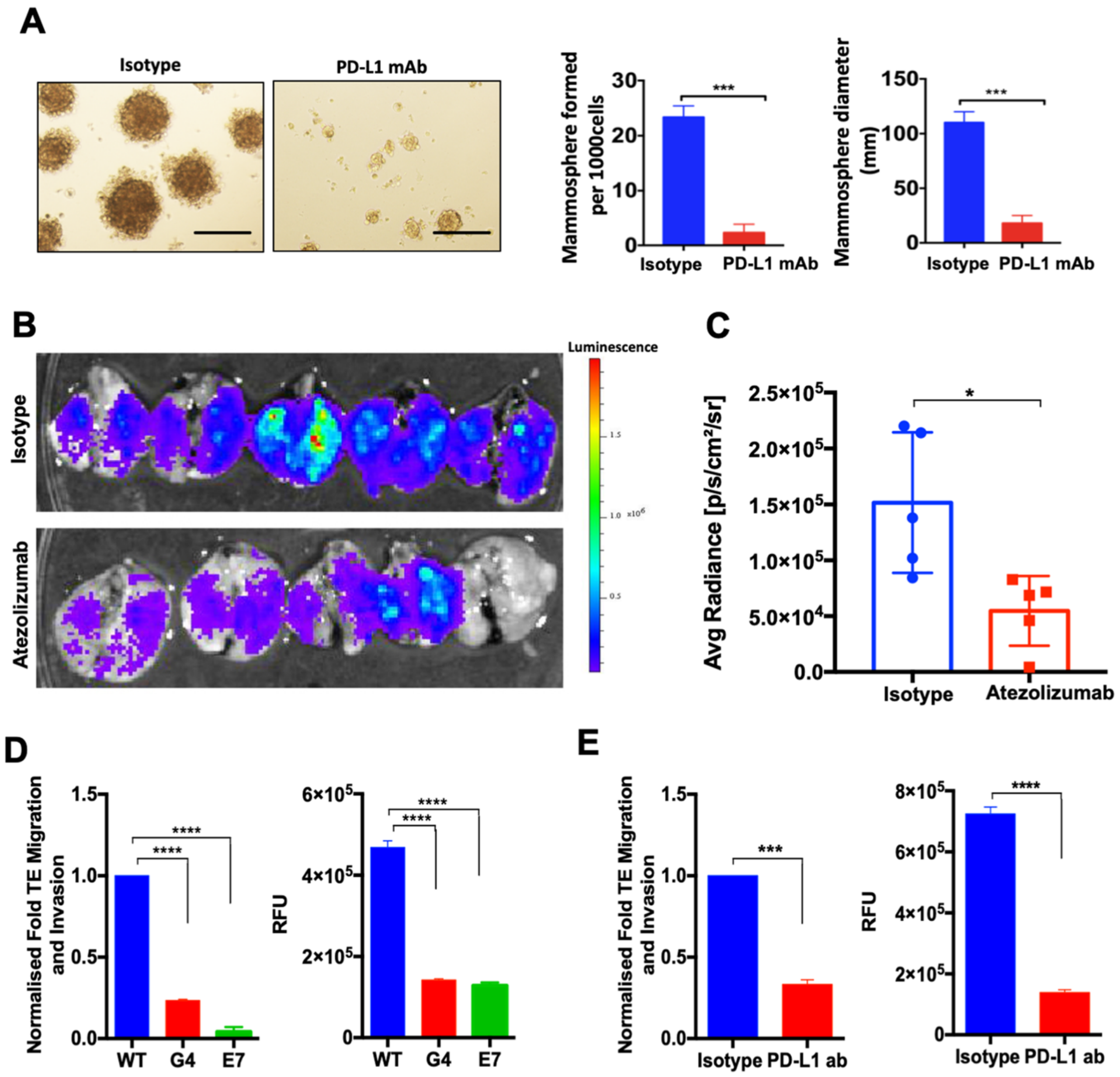

Figure S5: Anti-PD-L1 antibodies inhibit sphere formation by mammary cells, as well as transendothelial migration and metastasis of NSCLC cells, related to Figure 4. (**A**) MCF10A cells (1,000 cells per well) grown in mammosphere medium were seeded in ultra-low attachment plates in the presence of an anti-PD-L1 antibody (durvalumab; 0.05 mg/ml) or an isotype matched antibody. Photographs were captured two weeks later and spheres larger than 60 μm in size were measured and counted. (**B**) PC9ER cells (1X10^6) were injected into the tail vein of NSG mice. Forty-eight hours later, mice were randomized: one group (n=5) was intraperitoneally injected

with atezolizumab (0.2 mg per mouse) and the other received a control antibody (isotype matched). Injections were repeated on days 3, 6, 9, 12, 14, 16 and 18. On day 20, mice were treated with luciferin and 5 minutes later their lungs were resected. Lungs were visualized using the IVIS Spectrum In Vivo Imaging System (Perkin Elmer). (**C**) Quantification of the radiance signals from B. (**D**) Migration was assayed in Corning FluoroBlok multiwell insert plates with 8.0 μm pores. Prior to seeding in the insert, the apical side of the insert was coated with a basement membrane preparation (10 μg/ml, growth factor reduced). Human vascular endothelial cells (HUVECs; $5X10^4$ /well) were added into the coated inserts. EGF (30 ng/ml) was added in the base chamber as a chemo-attractant. After 24 hours, $4X10^4$ CellTracker Green CMFDA-labeled PC9ER or PD-L1 KO cells (G4 and E7) were added. After 22 additional hours, transendothelial migrated and invaded cells were imaged, and fluorescence intensity was quantified. (**E**) The protocol used in D was repeated except that cells were pretreated for 48 hours with an anti-PD-L1 antibody (durvalumab; 50μg/ml). Thereafter, the cells were added into the inserts in the presence of the antibody.

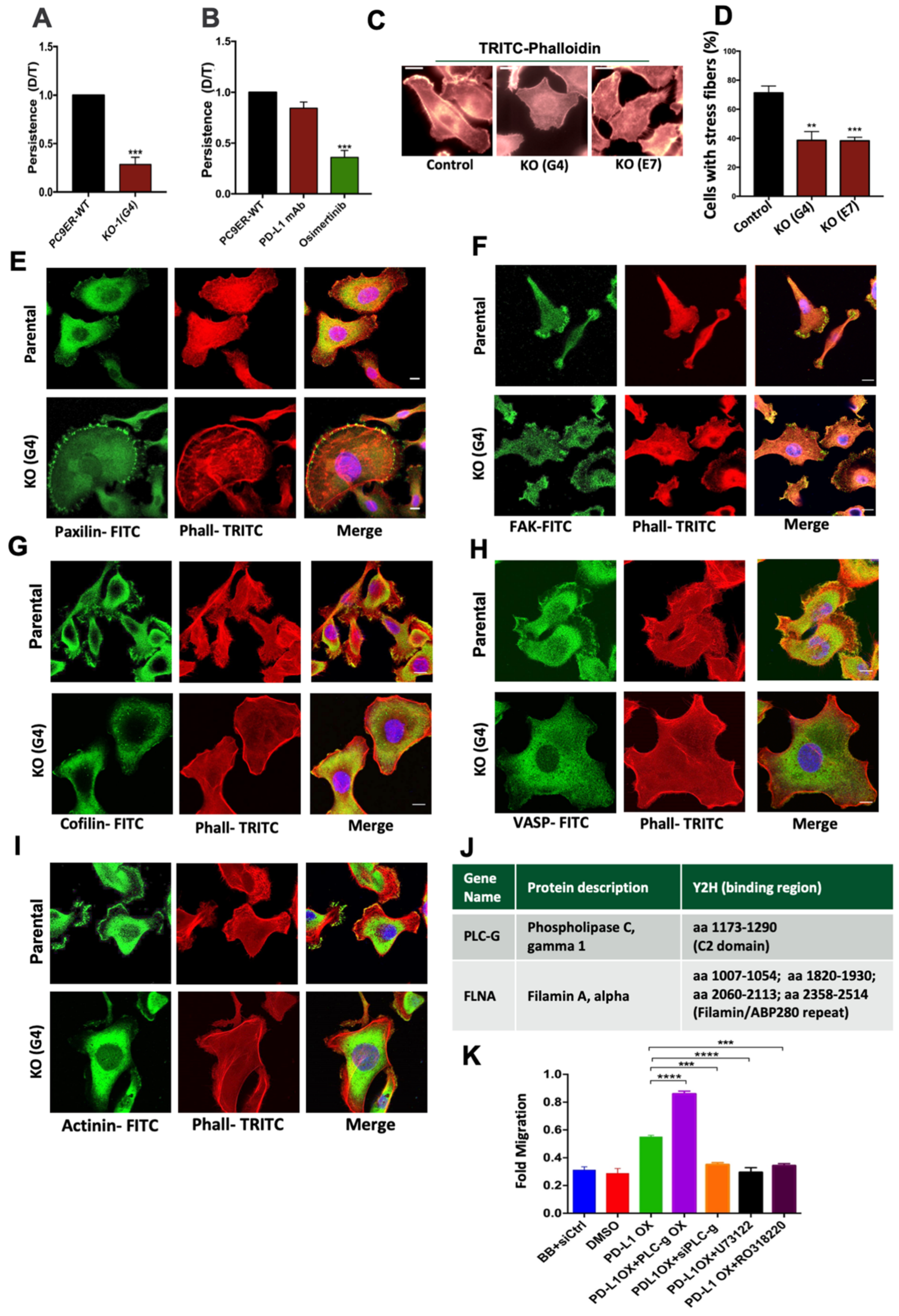

Gene Name	Protein description	Y2H (binding region)
PLC-G	Phospholipase C, gamma 1	aa 1173-1290 (C2 domain)
FLNA	Filamin A, alpha	aa 1007-1054; aa 1820-1930; aa 2060-2113; aa 2358-2514 (Filamin/ABP280 repeat)

Figure S6: PD-L1 regulates migration persistence and lamellipodia formation, likely by recruiting PLC-g1 and FLNA, related to Figure 5. (**A** and **B**) Chemotaxis assays of PC9ER and PD-L1-KO

cells were performed as described in Figs. 5A. Data from the respective rose plots were processed and scores of migration persistence were calculated by following migration of twenty cells per assay. (**C** and **D**) Cells ($0.5X10^6$) were seeded on coverslips and allowed to grow for 48 hours in media containing TGF-b (5 ng/ml). Samples were fixed with paraformaldehyde (4%), permeabilized with Triton-X (0.1%) and stained with Actin-stain 488 phalloidin (from Cytoskeleton Inc.). After mounting, the cells were imaged using a Nikon Eclipse Ti widefield microscope. Images were taken at 63X magnification and quantified in at least 6 non-overlapping fields of four independent experiments. (**E-I**) Control PC9ER cells ($0.5X10^6$) and cells lacking PD-L1 were seeded on coverslips and allowed to grow for 24 hours. Thereafter, cells were fixed and incubated overnight with antibodies specific to paxilin, FAK, cofilin, VASP and actinin. This was followed by a secondary, FITC-conjugated antibody. DAPI was used to visualize nuclei. Images were taken using confocal microscopy (63X magnification). Bars, 10 μM. (**J**) Yeast two-hybrid screens were performed using the portion of the human *PD-L1* cDNA that encodes the full-length cytoplasmic domain. This segment was cloned into a bait construct as an N-terminal fusion to LexA. The construct was used to screen a human lung cancer cDNA library and positive clones were selected. The respective prey fragments were amplified and sequenced. The resulting sequences were used to identify the corresponding interacting proteins. Listed are the two strongest interactors and their putative binding sites. (**K**) PC9ER cells were transfected with plasmids encoding PLC-g1 and PD-L1. Twenty-four hours later, some plates were transfected with siPLCg1, or siCtrl, and following 24 hours cells ($4X10^4$) were plated in the upper compartment of a 24-well Transwell tray. We used specific inhibitors of PLC-g1 (U73122) and PKC-a (RO 318220) at 2 μM throughout the assay, as indicated. The control group received DMSO alone. Cells were allowed to migrate for 18 hours through the intervening nitrocellulose filter (8-μm pore size). The filter was later removed and attached cells were fixed for 15 minutes in saline containing paraformaldehyde (4%). Cells located on the bottom side were photographed and quantified in triplicates (+ SEM). Bars, 500 μm

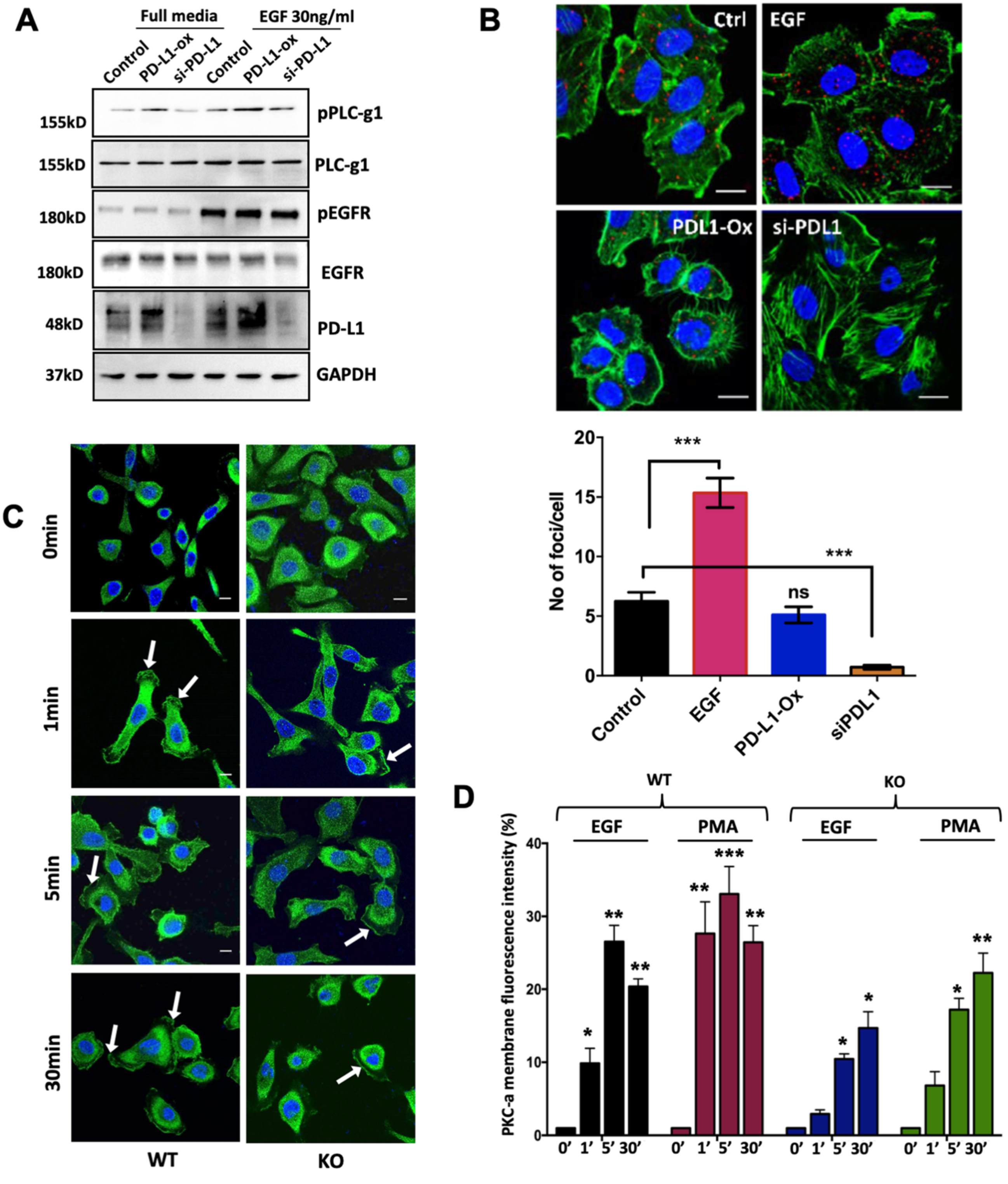

Figure S7: Cells depleted of PD-L1 are unable to activate PLC-g1 and its downstream signaling, related to Figure 5. (**A**) PC9ER cells were pre-transfected with siPD-L1 or with a plasmid encoding

PD-L1. Following 48 hours from transfection, cells were incubated with EGF and 30 minutes later their extracts were analyzed using the indicated antibodies. (**B**) PC9ER cells were pre-transfected with siPD-L1 or a plasmid encoding PD-L1, and 48 hours after transfection they underwent fixation. In parallel, parental PC9ER cells were untreated or treated for 30 minutes with EGF (30 ng/ml), prior to fixation. Similarly, cells lacking PD-L1 expression were fixed. Thereafter, all fixed cells were probed with antibodies recognizing PD-L1 and PLC-g1, and later processed for proximity ligation assays (PLA) that used a tetramethylrhodamine-5-isothiocyanate (TRITC) as a probe (red). Counterstaining used DAPI (blue) and phalloidin-FITC (green). The number of dots per cell were quantified using Image J (below panel). Scale bar, 10 µm (ns, not significant). (**C**) Parental PC9ER cells ($0.5X10^6$) and cells devoid of PD-L1 (KO) were seeded on coverslips. Cells were serum starved for 16 hours, and then treated with PMA (30 nM) for the indicated time intervals. Cells were washed in saline containing Tween 20, fixed and permeabilized. Thereafter, cells were fixed and incubated with a PKC-a specific antibody, followed by a secondary, FITC-conjugated antibody. Images were taken using confocal microscopy (63X magnification). Arrows mark PKC-a molecules recruited to the plasma membrane. Bars, 10 µm. (**D**) Cells were treated with EGF (30 ng/ml) or PMA (30 nM), and PKC-a recruitment to the plasma membrane was determined using immunofluorescence.
