# Supplementary material for: PD-L1 recruits phospholipase C and enhances tumorigenicity of lung tumors harboring mutant forms of EGFR
Source: Cell Rep. 2021 May 25;35(8):109181. doi: 10.1016/j.celrep.2021.109181 (PMC8170369; doi:10.1016/j.celrep.2021.109181)
Supplement: Document S2. Article plus supplemental information [file mmc4.pdf]

Cell Reports

Article

PD-L1 recruits phospholipase C and enhances tumorigenicity of lung tumors harboring mutant forms of EGFR

Graphical abstract

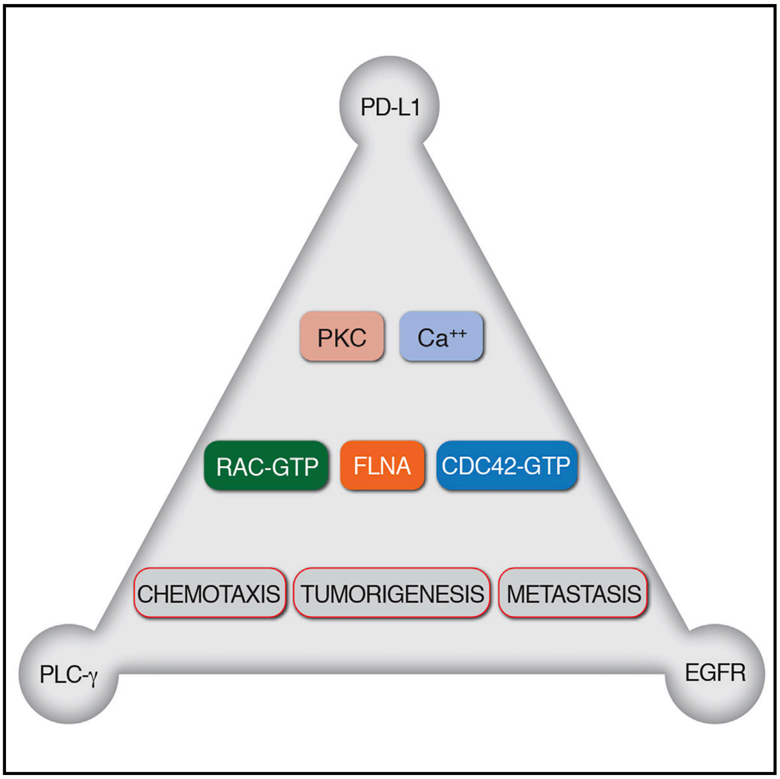

Highlights

- Unlike promoter-mediated PD-L1 induction by IFN-γ, EGFR rapidly stabilizes PD-L1 mRNA
- Once induced, PD-L1 enhances metastasis *in vivo* and chemotaxis toward EGF
- PD-L1 physically binds with and enhances activation of phospholipase C-γ1 by EGFR
- PLC-γ1 binds a PD-L1's cytoplasmic segment implicated in protection from cytotoxicity

Authors

Soma Ghosh, Nishanth Belugali Nataraj, Ashish Noronha, ..., Benjamin Geiger, Eytan Ruppin, Yosef Yarden

Correspondence

yosef.yarden@weizmann.ac.il

In brief

Unlike patients expressing mutant KRAS, patients with lung cancer harboring EGFR mutations are relatively resistant to immunotherapy. Because responses differ by EGFR allele, Ghosh et al. investigate the EGFR-to-PD-L1 axis. They report a three-way collaboration among EGFR, PD-L1, and phospholipase C-γ1. Targeting this triad may enhance responses to immunotherapy.

Ghosh et al., 2021, Cell Reports *35*, 109181
May 25, 2021 © 2021 The Author(s).
https://doi.org/10.1016/j.celrep.2021.109181

CellPress

Cell Reports

CellPress
OPEN ACCESS

Article

PD-L1 recruits phospholipase C and enhances tumorigenicity of lung tumors harboring mutant forms of EGFR

Soma Ghosh,[1,10] Nishanth Belugali Nataraj,[1,9] Ashish Noronha,[1,9] Sushant Patkar,[2,3] Arunachalam Sekar,[1] Saptaparna Mukherjee,[4] Sabina Winograd-Katz,[4] Lior Kramarski,[1] Aakanksha Verma,[1] Moshit Lindzen,[1] Diana Drago Garcia,[1] Joseph Green,[1] Galit Eisenberg,[5] Hava Gil-Henn,[6] Arkaprabha Basu,[7] Yan Lender,[8] Shimon Weiss,[7] Moshe Oren,[4] Michal Lotem,[5] Benjamin Geiger,[4] Eytan Ruppin,[3] and Yosef Yarden[1,11,*]

[1]Department of Biological Regulation, Weizmann Institute of Science, Rehovot, Israel
[2]Department of Computer Science, University of Maryland College Park, College Park, MD, USA
[3]Cancer Data Science Laboratory, National Cancer Institute, NIH, Rockville, MD, USA
[4]Department of Molecular Cell Biology Weizmann Institute of Science, Rehovot, Israel
[5]Sharett Institute of Oncology, Hadassah Medical School, Jerusalem, Israel
[6]The Azrieli Faculty of Medicine, Bar-Ilan University, Safed, Israel
[7]Department of Chemistry and Biochemistry, University of California, Los Angeles, Los Angeles, CA, USA
[8]The Shraga Segal Department of Microbiology, Immunology and Genetics, Ben-Gurion University of the Negev, Beer Sheva, Israel
[9]These authors contributed equally
[10]Present address: Division of Cancer Medicine, Department of Thoracic/Head & Neck Medical Oncology, MD Anderson Cancer Center, University of Texas, Houston, TX, USA
[11]Lead contact
*Correspondence: yosef.yarden@weizmann.ac.il
https://doi.org/10.1016/j.celrep.2021.109181

SUMMARY

Cancer immunotherapy focuses on inhibitors of checkpoint proteins, such as programmed death ligand 1 (PD-L1). Unlike RAS-mutated lung cancers, EGFR mutant tumors have a generally low response to immunotherapy. Because treatment outcomes vary by EGFR allele, intrinsic and microenvironmental factors may be involved. Among all non-immunological signaling pathways surveyed in patients' datasets, EGFR signaling is best associated with high PD-L1. Correspondingly, active EGFRs stabilize PD-L1 transcripts and depletion of PD-L1 severely inhibits EGFR-driven tumorigenicity and metastasis in mice. The underlying mechanisms involve the recruitment of phospholipase C-γ1 (PLC-γ1) to a cytoplasmic motif of PD-L1, which enhances PLC-γ1 activation by EGFR. Once stimulated, PLC-γ1 activates calcium flux, Rho GTPases, and protein kinase C, collectively promoting an aggressive phenotype. Anti-PD-L1 antibodies can inhibit these intrinsic functions of PD-L1. Our results portray PD-L1 as a molecular amplifier of EGFR signaling and improve the understanding of the resistance of $EGFR^+$ tumors to immunotherapy.

INTRODUCTION

Modern immunotherapy of cancer has focused on inhibitors of checkpoint proteins such as programmed death 1 (PD-1) and its ligand, PD-L1 (Sharma and Allison, 2015). Expression of PD-L1 on epithelial cells is induced by interferon-γ (IFN-γ), secreted by activated natural killer (NK) and T cells. Except for endothelial cells and the macrophage lineage, many human tissues only lowly express PD-L1. In contrast, PD-L1 is abundant in human carcinomas (Dong et al., 2002), as well as in the tumor microenvironment (TME) (Topalian et al., 2015). Constitutive expression of PD-L1 by tumor cells is frequently due to active oncoproteins, such as MYC, EML4-ALK, and AKT (Ota et al., 2015; Parsa et al., 2007), but how oncogenic forms of the epidermal growth factor receptor (EGFR) regulate the expression of PD-L1 is still incompletely understood. In an animal model, the expression of mutant EGFRs in bronchial cells can induce PD-L1 (Akbay et al., 2013), similar to EGF-stimulated esophageal squamous cell carcinoma (Zhang et al., 2017). In addition, treatment with an EGFR kinase inhibitor downregulated PD-L1 (Azuma et al., 2014), and EGFR-mediated upregulation of PD-L1 has been attributed to the ERK/Jun pathway (Chen et al., 2015). According to some recent lines of evidence, PD-L1 may have tumor-intrinsic oncogenic functions, beyond the evasion of anti-tumor immune responses. Thus, PD-L1 contributes to cancer stemness (Zhong et al., 2017) and the epithelial-mesenchymal transition (EMT) (Chen et al., 2014; Dong et al., 2018; Lou et al., 2016; Wang et al., 2018). Pathological studies of esophageal cancer demonstrated that tumor samples enriched with EMT markers had higher PD-L1 expression (Chen et al.,

This is an open access article under the CC BY license (http://creativecommons.org/licenses/by/4.0/).

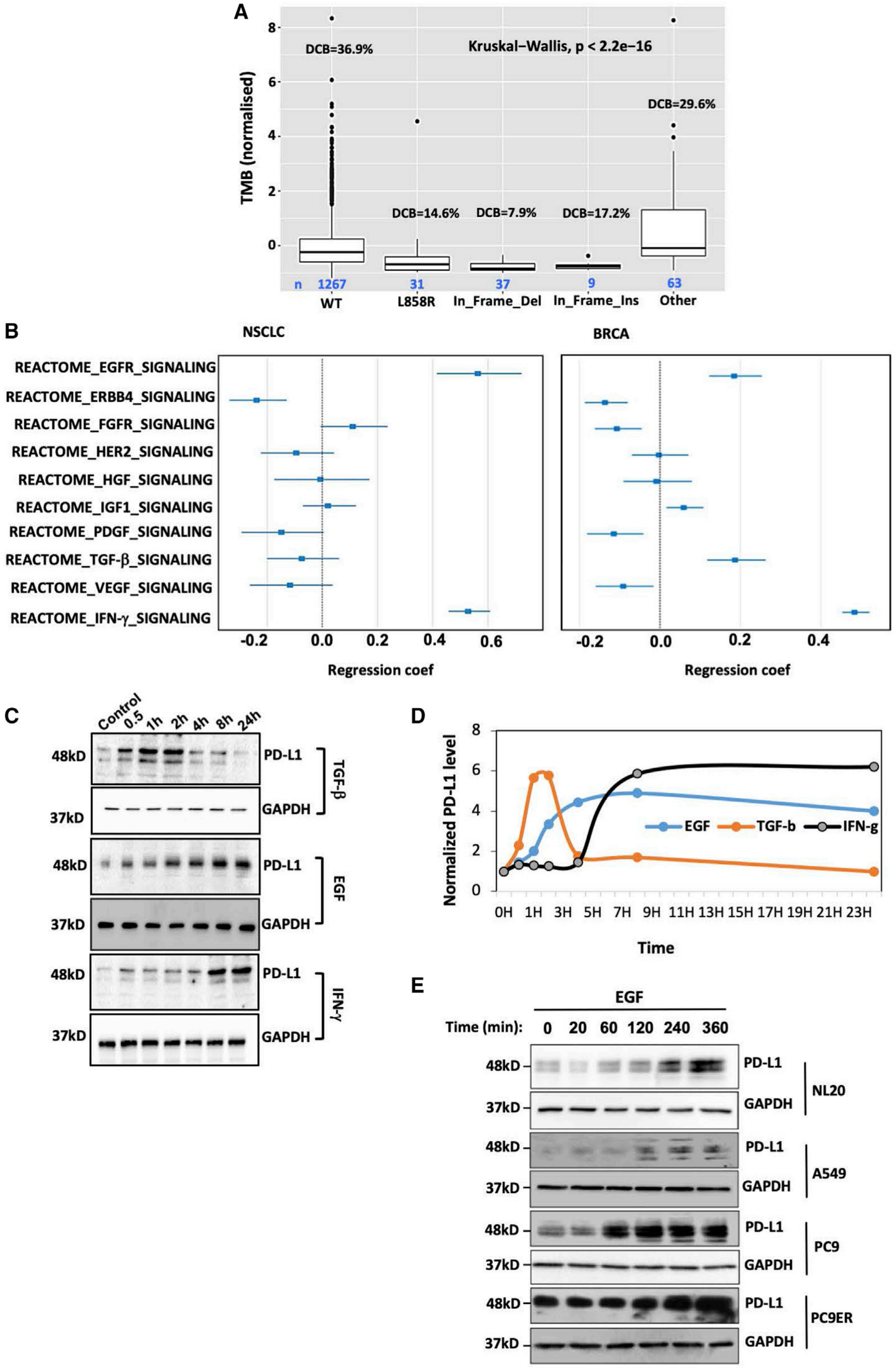

(legend on next page)

CellPress
OPEN ACCESS

2017). However, other reports demonstrated that PD-L1 regulates the sensitivity of cancer cells to cytotoxic agents (Li et al., 2017; Tu et al., 2019).

The studies presented herein were driven by reports demonstrating that EGFR mutations are associated with low response rates to PD-1 pathway blockade in non-small cell lung carcinoma (NSCLC) (Gainor et al., 2016). Importantly, patients with uncommon *EGFR* mutations were associated with relatively good responses to immunotherapy (Evans et al., 2020; Yamada et al., 2019). Likewise, another study concluded that treatment outcomes varied by EGFR allele (Hastings et al., 2019), implying that allele-specific attributes contribute to responses to immunotherapy. Our quest for non-immunological factors that frequently induce PD-L1 in lung tumors found that only one route, which is activated by EGFR, was strongly associated with PD-L1 abundance. Subsequent experiments linked the inducible PD-L1 molecules to chemotaxis and to both tumorigenesis and metastasis in animals. According to the emerging model, physical recruitment of phospholipase C-γ1 (PLC-γ1) to the cytoplasmic tail of PD-L1 enables active EGFRs to trans-phosphorylate and activate PLC-γ1, thereby instigating a cascade leading to an aggressive phenotype, which may underlie the resistance of *EGFR* mutant tumors to immunotherapy.

RESULTS

Relationships between EGFR mutations and patient response to immunotherapy implicate stromal inducers of PD-L1

Along with mismatch repair deficiency and PD-L1 expression, well-established predictors of response to immune checkpoint blockers (ICBs), several studies have shown that greater tumor mutation burden (TMB) is associated with a greater likelihood of response of NSCLC to immunotherapy (Rizvi et al., 2015). Consistent with the relatively high TMB of the *KRAS* mutant subgroup of patients, ICBs better prolonged overall survival in this subgroup, as compared to the *KRAS* wild-type (WT) subgroup (Lee et al., 2018). However, the same treatments prolonged overall survival in the *EGFR* WT subgroup, while inducing only weak improvements in the *EGFR* mutant subgroup. In addition, the exact type of EGFR mutation seems to affect response (Yamada et al., 2019). These observations propose that additional factors, other than TMB, influence the response of $EGFR^+$ tumors. To better understand the relationships between EGFR mutations, TMB, and patient response, we pooled data from 1,523 patients (Hellmann et al., 2018; Rizvi et al., 2015, 2018). Only patients with NSCLC who received anti-PD-1 or anti-PD-L1 monotherapy were considered. TMB was mean centered, and durable clinical benefit (DCB) summed partial response/complete response and stable disease (progression-free survival [PFS] > 6 months). Figure 1A presents the results in terms of TMB, DCB, and EGFR mutations. While tumors expressing WT or uncommon forms of EGFR displayed relatively high DCB, patients with other mutant forms, especially in-frame deletions, displayed low DCB and apparent discordant relationships between TMB and DCB. Hence, we performed a log likelihood ratio analysis comparing two competing parameters, TMB and EGFR status. This analysis revealed that EGFR mutation status can explain differences in clinical outcome above and beyond what is already explained by TMB ($p = 0.01104$). Conceivably, intrinsic attributes, such as autocrine loops or coupling of specific EGFR mutations to downstream pathways, may change the DCB.

The TME may contribute to sensitivity to immunotherapy. Hence, we analyzed two datasets of RNA sequences derived from patients with lung adenocarcinoma (1,017 patients). A multivariate regression analysis was performed to assess unique contributions of signaling pathways. Expression values were transformed to log TPM (total productive maintenance) and later normalized (Figure 1B). In addition to IFN-γ, we focused on all growth factor pathways previously implicated in the progression of epithelial tumors (Witsch et al., 2010). This analysis revealed that the EGFR pathway, more than the other routes, was associated with PD-L1, and this was not biased by the small fraction of tumors with EGFR mutations. To examine the possibility that our finding was relevant to other cancers, we analyzed breast cancer (1,093 patients). Once again, the IFN-γ and EGFR pathways strongly contributed to PD-L1 expression, but unlike lung tumors, the transforming growth factor-β (TGF-β) pathway emerged as a secondary player (Figure 1B). To experimentally verify these observations, we used non-transformed MCF10A mammary epithelial cells. As predicted, not only IFN-γ but also EGF and TGF-β strongly upregulated PD-L1 in MCF10A cells (Figure 1C). However, the kinetics of induction differed: the effect of TGF-β peaked at 1–2 h and the later onset of induction by EGF preceded the increase induced by IFN-γ (Figure 1D).

Presumably, kinase-activating EGFR mutations, which highly increase PD-L1 expression in lung cells (Akbay et al., 2013; Azuma et al., 2014), mimic stimulation by EGF. To experimentally verify this model, we tested normal lung cells (NL20). Stimulation with EGF gradually increased PD-L1 levels (Figure 1E). Similar

Figure 1. EGFR mutation-specific durable clinical benefit proposes intrinsic determinants of response to immune checkpoint inhibitors (ICIs) and transcriptome analyses identify EGFR as the major nonimmunological driver of PD-L1

(A) TMB was calculated for the indicated alleles of EGFR from 5 datasets and the p value was calculated using the Kruskal-Wallis test. All of the values were mean centered and normalized. The number of patients per group and durable clinical benefit (DCB) from anti-PD-1 or anti-PD-L1 monotherapies are indicated. Statistics were calculated using the log likelihood ratio test ($p < 0.01$).

(B) Regression analysis was performed on the following RNA sequencing (RNA-seq) datasets: left panel (lung cancer): The Cancer Genome Atlas (TCGA) lung adenocarcinoma (LUAD) ($n = 516$) and TCGA lung squamous cell carcinoma (LUSC) ($n = 501$). Right panel (breast cancer): TCGA-breast cancer gene (BRCA) datasets of 1,093 patients with breast cancer. Following integration of the datasets, we transformed the expression values to log TPM and normalized. p value, $1.14e{-}14$.

(C) MCF10A cells were stimulated with EGF, TGF-β, or IFN-γ (each at 30 ng/mL), harvested at the indicated time points and extracts were probed.

(D) Band intensities from (C) were quantified and normalized to glyceraldehyde 3-phosphate dehydrogenase (GAPDH).

(E) The indicated NSCLC cell lines, along with the non-cancerous NL20 cells (3×10^6), were incubated with EGF (30 ng/mL) and extracted at the indicated time points.

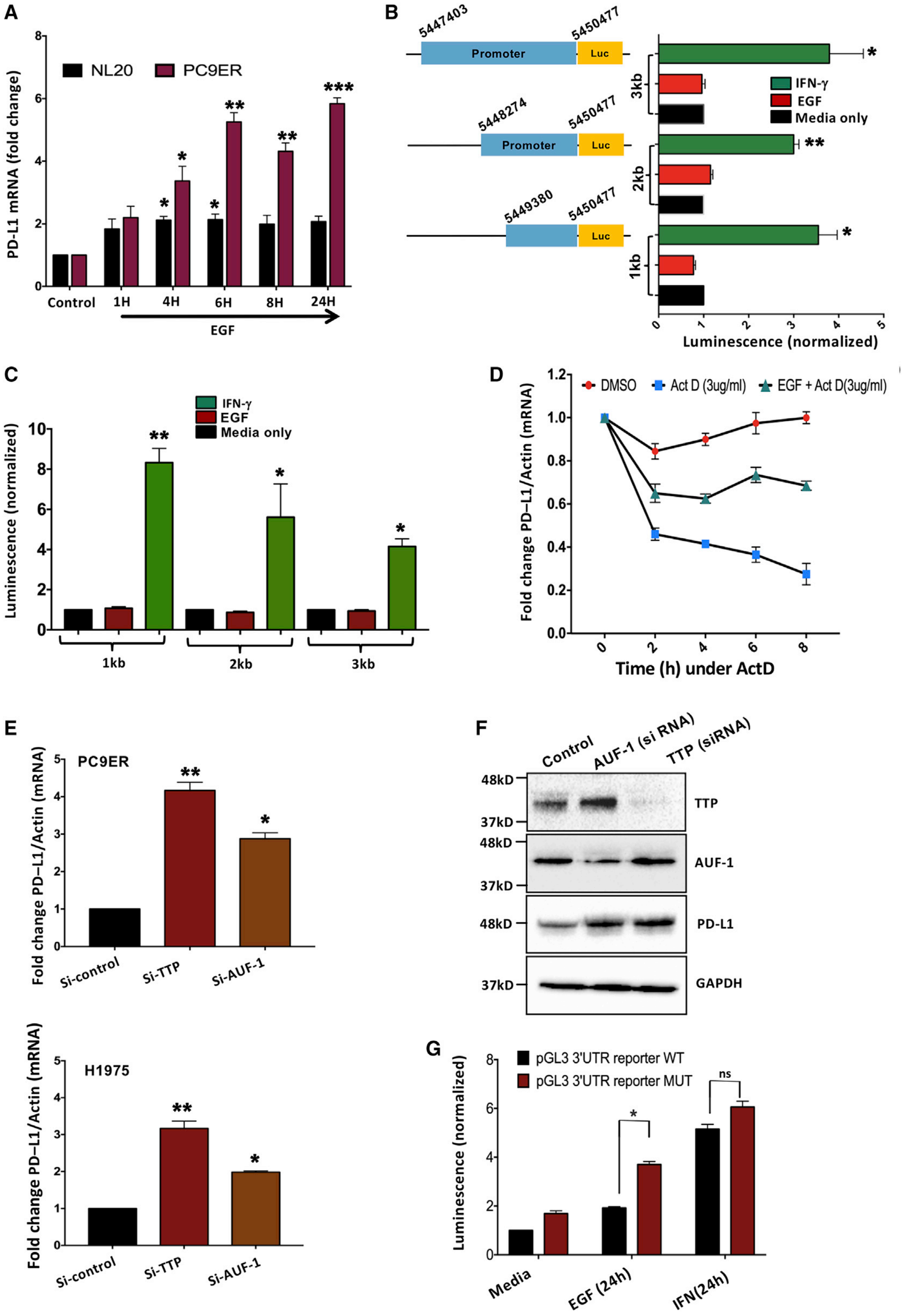

Figure 2. EGFR signaling increases PD-L1 mRNA stability

(A) The indicated cell lines were treated with EGF and qPCR was performed to determine levels of the mRNA of PD-L1. Signals were normalized to actin transcripts.

(legend continued on next page)

patterns were displayed by PC9 cells (del 746-750-EGFR) and the derivative PC9ER cells, expressing in addition a secondary mutation, T790M (Figure 1E). Concentrating on PC9ER cells and contrasting EGF and IFN-γ, we verified the kinetic differences and noted that IFN-γ rapidly activated STAT-3, whereas EGF activated ERK (Figure S1A). Furthermore, using cytometry and immunofluorescence, we validated surface localization of the inducible PD-L1 molecules (Figures S1B and S1C). Similar observations were made with H1975 cells (L858R and T790M mutations) and WT EGFR expressing A549 cells. To directly link EGFR mutations to PD-L1, we expressed in NL20 cells mutant forms of EGFR. This isogenic system showed that the mRNA and protein levels of PD-L1 correlated with the strength of the auto-phosphorylation of EGFR (Figures S1D and S1E). In summary, analyses of tumors with EGFR mutations uncovered mutation-specific discordance between TMB and response to immunotherapy. This led us to analyze stromal factors and identify EGFR signaling as the major non-immunological pathway that increases PD-L1 abundance.

While IFN-γ induces promoter activation, EGF stabilizes the transcripts of PD-L1

Interferon regulatory factor 1 (IRF1), a transcription factor regulated by IFNs, binds with the PD-L1 promoter following the activation of the JAK/STAT axis (Garcia-Diaz et al., 2017). In contrast, it has been reported that RAS stabilizes the mRNA of PD-L1 (Coelho et al., 2017), while EGF stabilizes PD-L1 via glycogen synthase kinase 3β (GSK3β) inactivation (Li et al., 2016). Analyses performed on RNA isolated from NL20 and PC9ER cells confirmed EGF-dependent mRNA upregulation (Figure 2A). Because this analysis cannot distinguish newly synthesized from stabilized transcripts, we used promoter reporters. The results confirmed that treatment with IFN-γ activated new transcription. However, stimulation with EGF was ineffective (Figures 2B and 2C). Hence, we inhibited RNA synthesis using actinomycin D (Figure 2D). Co-treatment with actinomycin D and EGF significantly retarded the rate of decay of PD-L1 RNA, in line with the prediction that EGF can stabilize the mRNA of PD-L1. The 3′ untranslated region (3′ UTR) of the transcripts of PD-L1 contains several AU-rich elements (AREs), which recruit ARE binding proteins (AUBPs) able to enhance mRNA decay. Hence, we transfected cells with small interfering RNAs (siRNAs) targeting two major AUBPs, TTP and AUF-1. As predicted, depletion of the respective mRNAs enhanced the transcripts of PD-L1 (Figure 2E) and protein (Figure 2F). Next, we transfected cells with two luciferase reporters: a WT reporter containing six ATTTA pentamers from the 3′ UTR of the mRNA of PD-L1, and a construct containing six copies of a defective pentamer (ATGTA) (Rajagopalan et al., 1995). While treatment with IFN-γ induced a statistically insignificant difference between the reporters, in cells prestimulated with EGF, the mutant reporter yielded higher signals (Figure 2G). In conclusion, unlike the effect of IFN-γ, EGF elevates the respective transcript by means of prolonging the mRNA half-life.

Overexpression of PD-L1 accelerates tumor growth and enhances both invasiveness and cell proliferation

Next, we overexpressed a PD-L1-GFP fusion protein and confirmed high expression (Figure 3A). PD-L1-overexpressing cells placed in Transwells migrated more rapidly, as compared to control cells (Figure 3B). Coating the filter with a preparation of a basement membrane indicated that PD-L1-GFP also conferred enhanced matrix invasion (Figure 3C). Notably, cell motility requires actin-based plasma membrane protrusions. Hence, we expressed GFP-PD-L1 in HeLa cells and recorded live cell images. Surprisingly, PD-L1-overexpressing cells displayed remarkably abundant and long filopodia (Figure 3D). Next, we applied a radioactive thymidine incorporation assay. The results indicated that PD-L1 overexpression doubled the incorporation of thymidine into DNA (Figure 3E). Next, we collected conditioned media for cytokine array analysis. The most remarkable difference we observed entailed the upregulation of interleukin-18 (Figure S2A). Notably, an immunohistochemical analysis of pulmonary adenocarcinoma found that PD-L1 was positively correlated with EMT markers (Kim et al., 2016). Hence, we applied PCR (Figure 3F) and immunoblotting (IB) (Figure 3G) and verified that PD-L1 overexpression increased SNAIL, SLUG, and ZEB1, and decreased several adhesion molecules. To examine effects on tumorigenesis, we established overexpressing H1975 cells and subcutaneously implanted them in athymic mice. While control cells slowly developed tumors, the PD-L1-overexpressing subline displayed significantly more robust tumorigenesis (Figures 3H and 3I). These observations demonstrated that high PD-L1 confers accelerated proliferation rates, along with tumorigenesis and several features of invasive phenotypes.

Depletion of PD-L1 inhibits cell-cycle progression, reduces invasiveness, and enhances cell adhesion

To resolve the modes of action of PD-L1 in NSCLC cells, we undertook several loss-of-function approaches. First, by using CRISPR-Cas9 procedures (Ran et al., 2013), we ablated the

(B and C) PC9ER cells were transfected with the indicated reporters corresponding to human *PD-L1*. Plasmids containing different length inserts of genomic DNA lying upstream to the initiator codon of the *PD-L1* gene were used. Cells were treated for 8 (B) or 24 h (C) with medium only, EGF (30 ng/mL), or IFN-γ (10 ng/mL). The numbers correspond to the region selected from the human reference genome assembly GRCh38. The normalized data (averages ± SEs of triplicates) are representatives of 3 independent experiments.

(D) PC9ER cells were co-treated with EGF (or vehicle), actinomycin D, or the respective combination, and qPCR analysis was used to determine the transcript levels of PD-L1.

(E) PC9ER and H1975 cells were transfected with siRNAs targeting AU-rich element binding proteins, or with siScrambled (siSc). Cells were analyzed 48 h later for PDL1 transcript (in triplicates).

(F) PC9ER cells were transfected with siRNAs as in (E). Extracts were analyzed 48 h later using immunoblotting (IB).

(G) PC9ER cells were starved overnight and later transfected with luciferase reporter plasmids containing wild-type $(ATTTA)_6$ or mutant $(ATGTA)_6$ versions of the PD-L1 3′ UTR. Luciferase signals were determined 24 h after transfection. Data depict the normalized means ± SEs.

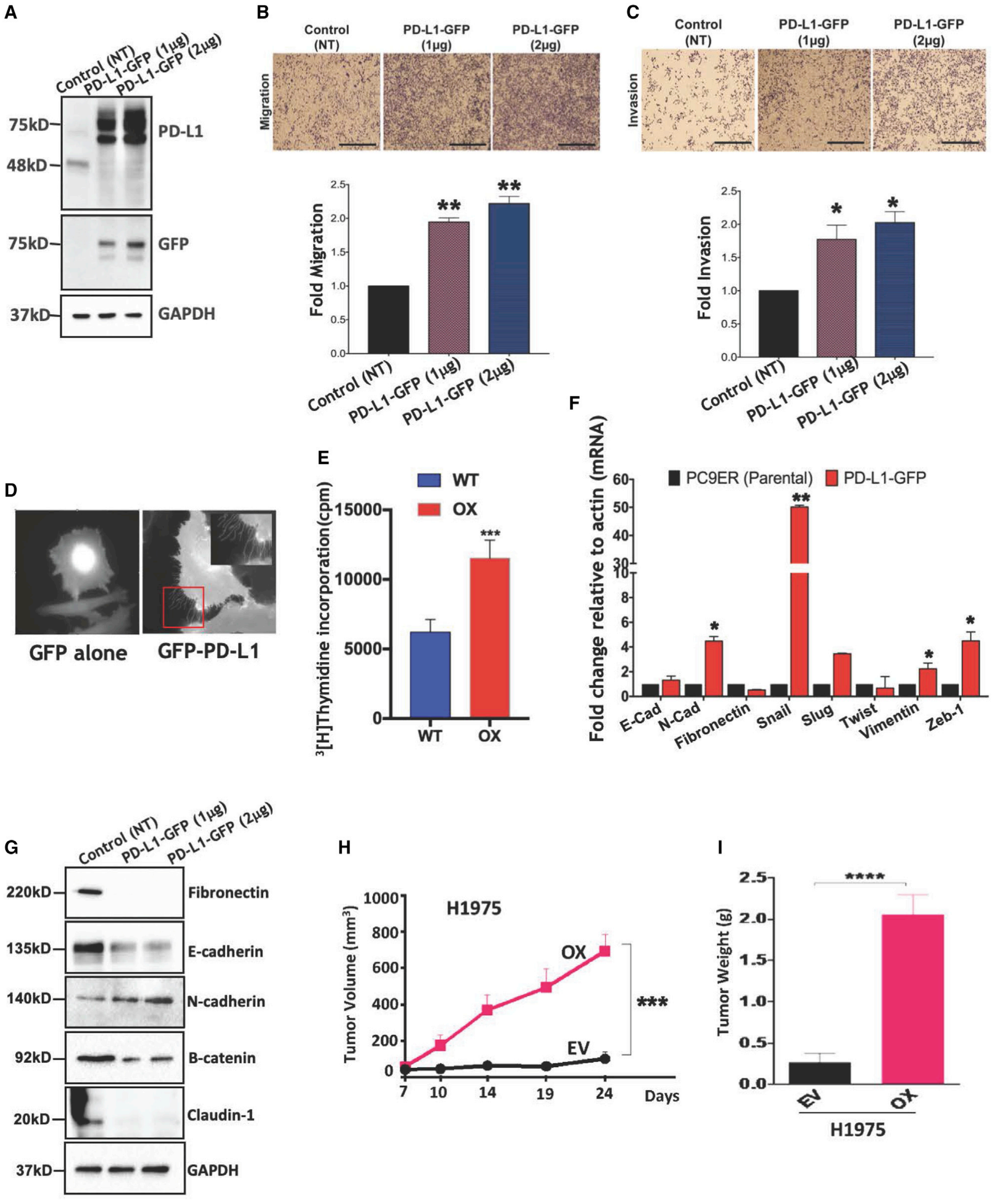

Figure 3. High expression of PD-L1 instigates an invasive phenotype and enhances tumorigenesis

(A) PC9ER cells and cells pretransfected with a plasmid encoding a PD-L1-GFP fusion protein (or GFP alone; *NT*) were extracted 48 h after transfection. Extracts were immunoblotted.

(legend continued on next page)

expression of PD-L1 in PC9ER cells and selected two clones (Figure 4A). A comparison of proliferation rates indicated that the depletion of PD-L1 inhibited proliferation (Figure S2B). This may be due to the reduced secretion of growth factors, since we detected lower levels of vascular endothelial growth factor (VEGF) and two EGFR ligands in media conditioned by a knockout (KO) clone (Figure S2C). In addition, the KO clones displayed significantly reduced rates of migration (Figure S2D). To verify the phenotype, we knocked down PD-L1 using both siRNAs and small hairpin RNAs (shRNAs), and re-assayed cell migration and matrix invasion. The results established a link between low PD-L1 and retarded motility (Figures S2E–S2I). Importantly, the observed effects of shPD-L1 were reproducible when two other lines were tested: H1299 (WT EGFR) and H1975 (Figures S3A and S3B). The tests performed with these cells reinforced the link between lowered abundance of PD-L1 and retarded rates of migration (Figures S3C and S3D) and proliferation (Figures S3E and S3F). These results confirmed all of the observations we made while using PC9ER cells.

Second, we performed live imaging of gap closure with KO cells and observed a >50% reduction in the average cell front velocity (Figure 4B). Congruently, seeding KO cells in plates that were precoated with fibronectin revealed increased propensity to adhere to the substrate (Figure 4C). Remarkably, these cells displayed ~10-fold slower rates of DNA synthesis (Figure 4D), and determination of cell-cycle distributions revealed that the loss of PD-L1 increased the fraction of cells found in G2/M (Figure 4E). Further analysis detected increased levels of fibronectin, claudin-1, and β-catenin, as well as reduced vimentin and N-cadherin (Figure 4F). In conclusion, PD-L1 propels both migration and proliferation of $EGFR^+$ lung cancer cells and reduces cell-to-cell and cell-to-matrix adhesion.

PD-L1 is essential for growth factor-inducible migration and for tumorigenesis and metastasis

As expected, both EGF and TGF-β strongly increased the migration of PC9ER cells, but IFN-γ exerted no motility effect (Figures 4G and 4H). Importantly, KO cells displayed lower basal migration, and their responses to EGF and TGF-β were weaker. It is interesting to note that tumor aggressiveness and patient survival correlate with migratory properties of cultured cancer cells (Nair et al., 2019). Hence, we predicted that the loss of PD-L1 would retard not only tumorigenic growth but also metastatic spread. To test this, we subcutaneously inoculated PC9ER cells, along with a KO derivative, in two strains of immunocompromised mice. The results revealed a major defect in tumorigenic growth of the KO cells in both strains (Figures 4I and 4J). In a similar way, transplanting shPD-L1 expressing H1975 and H1299 cells confirmed that cells in which PD-L1 was downregulated displayed retarded tumor growth (Figures S3G and S3H). Because of the major growth differences, we applied tail vein rather than orthotopic metastasis assays; luciferase-labeled KO cell derivatives were established and injected into the tail vein of NSG mice. Unlike lungs isolated from control mice 10 days later, which were densely populated by metastatic nodules, none of the mice preinjected with the KO cells developed detectable metastases (Figures 4K and 4L). These effects raised the possibility that defects in motility and proliferation, which are characteristic of the KO cells, were responsible for the complete inhibition of metastasis. Hence, we probed thin sections of excised lungs for Ki67 and human cytokeratin 18 (CK18). Unlike WT cells, which invaded the parenchyma and displayed Ki67 positivity (Figures S4A and S4B), PD-L1-ablated cells were confined to the rims of bronchi and displayed relatively weak proliferation. Our observations indicated that PD-L1 is strictly essential for robust tumor growth and metastasis in animal models.

Clinically approved anti-PD-L1 antibodies inhibit growth of lung cancer cells, retard trans-endothelial migration (TEM), and inhibit metastasis

Immune checkpoint inhibitors (ICIs) targeting PD-L1 have shown clinical efficacy against different malignancies (Akinleye and Rasool, 2019). Assuming that durvalumab, a clinically approved anti-PD-L1 antibody, blocks the previously described (Clark et al., 2016; Gato-Cañas et al., 2017) and herein extended cell-autonomous functions of PD-L1, we analyzed the effects on ERK and AKT. The results uncovered antibody-dependent downregulation of the PD-L1 protein (Figure S4C). Notably, a similar assay that used MDA-MB-231 cells observed downregulation by H1A, an anti-PD-L1 antibody, but durvalumab was ineffective in their assays (Tu et al., 2019). Concomitant with downregulation, the activated (phosphorylated) form of AKT was decreased. In addition, durvalumab inhibited cell migration (Figure S4D) and inhibited the incorporation of radioactive thymidine (Figure S4E). Next, we used non-transformed MCF10A mammary cells, which migrate in response to growth factors by activating AKT2 (Irie et al., 2005). We verified that PD-L1 depletion using siRNAs could retard EGF and TGF-β-induced migration (Figure S4F). As expected, durvalumab partly inhibited the motogenic activity of both EGF and TGF-β, while a control antibody was inactive (Figure S4G).

It is notable that MCF10A cells can form mammospheres, which recapitulate features of the glandular epithelium (Debnath and Brugge, 2005). Hence, cells were incubated with durvalumab in ultra-low attachment plates, and 2 weeks later, they were photographed. The results demonstrated strong inhibitory

(B and C) PD-L1-GFP-overexpressing PC9ER cells (4×10^6) were assayed in Transwell (B) or Matrigel-coated chambers (C). Paraformaldehyde was used to fix cells that reached the lower side of the intervening filter. Cell images and average signals are shown.

(D) HeLa cells were transfected with a vector encoding PD-L1 fused to GFP or GFP only. Shown are frames from live cell videos captured 36 h later. The framed area is enlarged in the upper right corner.

(E) PC9ER cells pretransfected with either a pGIPZ-EGFP plasmid or with the pGIPZ-PD-L1-EGFP plasmid were analyzed. ^{3}H-thymidine (1 μCi) was added after 16 h, and radioactivity incorporated into DNA was measured 48 h later. Shown are averages ± SDs of quadruplicates.

(F and G) Control PC9ER cells or cells transiently overexpressing PD-L1-GFP were analyzed using either qPCR (F) or IB (G).

(H and I) PD-L1-overexpressing H1975 cells (1×10^6) or cells transfected with an empty vector (EV) were implanted in nude mice (n = 6). Tumor volumes were estimated once per week. At the end of the experiment, tumors were excised and their weights were determined (right panel; averages ± SEMs).

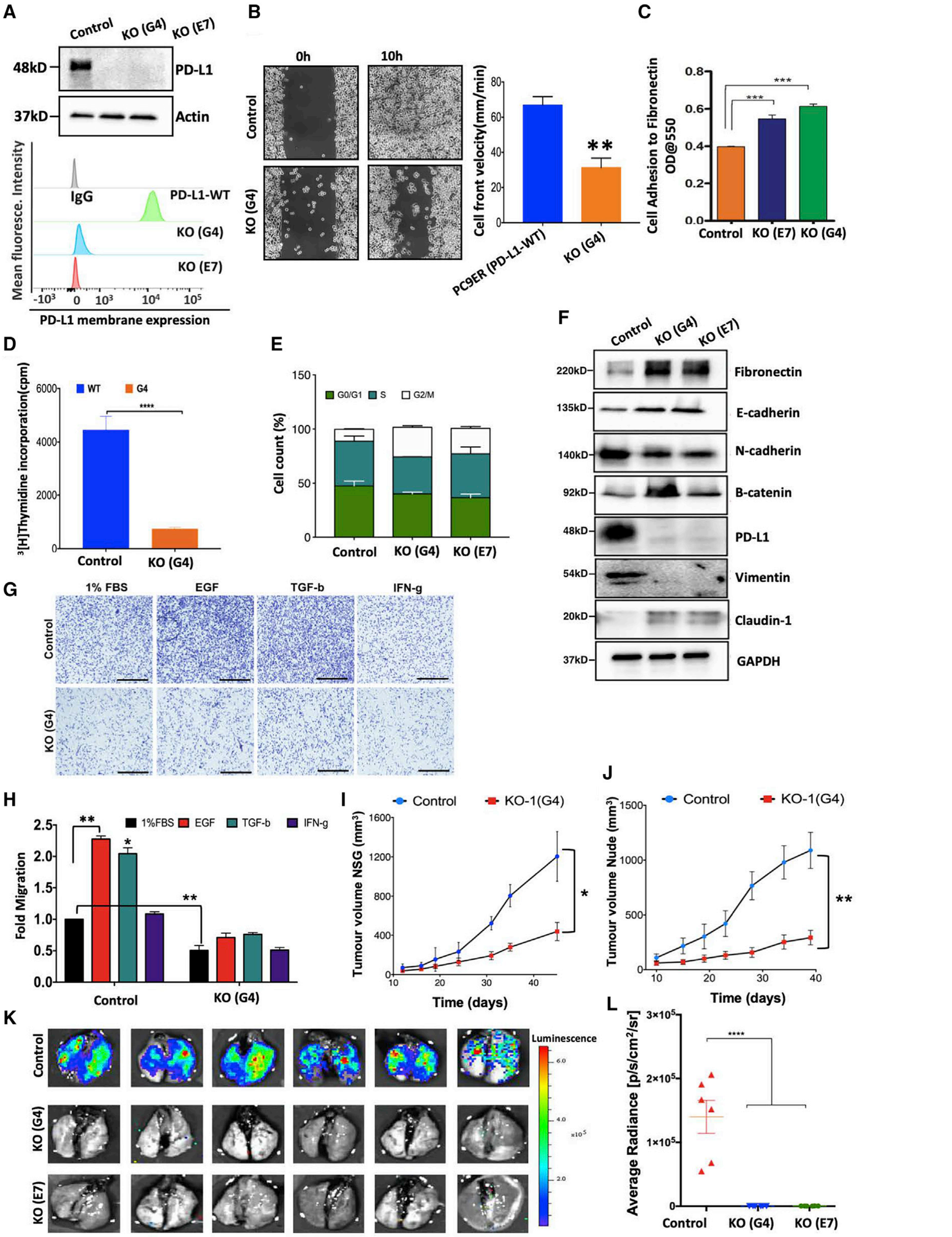

Figure 4. Loss of PD-L1 enhances adhesion and inhibits proliferation and migration, as well as tumorigenesis and metastasis
(A) Extracts from parental and PD-L1 KO clones were analyzed using IB. The lower panel presents cytometry results obtained using antibodies recognizing PD-L1.

(legend continued on next page)

effects (Figure S5A). To extend this to metastasis, we injected luciferase-labeled PC9ER cells into the tail vein of NSG mice. Two groups of mice were intraperitoneally injected with atezolizumab, another clinically approved anti-PD-L1 antibody. Injections were repeated twice per week, and on day 20 mice were sacrificed. Analyses of the lungs indicated a statistically significant inhibition of metastasis (>60%; Figures S5B and S5C). While this format reflects extravasation, entry of cancer cells into the circulation may be reliably examined using TEM assays. Accordingly, we seeded human vascular endothelial cells (HUVECs) in fluoro blocks precoated with a basement membrane. PC9ER (WT) and KO cells were added, and cells that migrated across the barrier were quantified (Figure S5D). The results revealed major differences between WT and KO clones. Using the same setting, WT cells were pretreated with durvalumab and later they were used to overlay HUVEC-containing inserts in the presence of durvalumab (Figure S5E). The results demonstrated that the antibody strongly inhibited TEM (>60%). These observations attribute to PD-L1 hitherto unrecognized roles in TEM and metastasis of $EGFR^+$ lung cancer and imply that antibodies such as durvalumab can inhibit tumor progression independently of immune interactions.

PD-L1 regulates Rho family GTPases and enhances directional persistence of migration

Next, we created a gradient of EGF and measured directionality, which relates the linear distance between the start and endpoints (D) to the total distance (T) traveled. The results indicated that the directional persistence (D/T) of cells lacking PD-L1 was severely reduced (Figures 5A and S6A), and pretreatment with durvalumab or osimertinib, an EGFR kinase inhibitor, reduced persistence (Figures 5B and S6B). The switch controlling random versus persistent migration involves actin filaments (Pegtel et al., 2007) and Rho family GTPases (Etienne-Manneville and Hall, 2003; Nobes and Hall, 1999; Petrie et al., 2009). Specifically, RAC promotes the formation of peripheral lamellae, which mediate random migration (Pankov et al., 2005), but also contributes to persistent migration (Petrie et al., 2009). To assay Rho proteins, we used the ability of effectors to bind with the GTP-bound forms of small GTPases. The assay indicated that ablation of PD-L1 reduced CDC42-GTP and RAC1-GTP, but increased RHOA-GTP (Figure 5C). Congruently, more active RAC1 and CDC42 were detected in PD-L1-overexpressing cells (Figure 5D). Because ablation of PD-L1 increased substrate adhesion and Rho family members regulate adhesion and stress fibers (Nobes and Hall, 1999), we visualized actin in KO cells. The results reflected the severe loss of actin cables (Figures S6C and S6D). In line with this observation, we noted the redistribution of the focal adhesion kinase (FAK) and paxillin, as well as loss of lamellipodial structures, which were visualized using antibodies to different cytoskeletal proteins (Figures S6E–S6I). In light of the ability of Rho family GTPases to control directional migration (Petrie et al., 2009), our findings may explain the association between an overexpressed-PD-L1, directional persistence of migration and metastasis.

The cytoplasmic domain of PD-L1 physically binds with both Filamin A (FLNA) and PLC-γ1

Conceivably, the cytoplasmic tail of PD-L1 enables direct interactions with downstream signaling proteins controlling the actin cytoskeleton. In line with this prediction, mutant forms of the tail exist in carcinomas (Gato-Cañas et al., 2017), and putative interactors have been identified (Huttlin et al., 2015). To uncover direct binders, we performed yeast 2-hybrid screens. The cytoplasmic tail of PD-L1 was cloned into a bait construct and >50 million interactions were screened (Fromont-Racine et al., 1997). Two PD-L1 interaction partners reached the highest confidence score: FLNA and PLC-γ1 (Figure S6J). FLNA is a dimeric protein supporting oncogenic phenotypes (Shao et al., 2016). Each monomer comprises an actin-binding domain, which anchors actin fibers to each other, thereby controlling cell rigidity. The other candidate, PLC-γ1, acts as a RAC1 guanine nucleotide exchange factor that regulates EGF-induced cell migration (Li et al., 2009). PLC-γ1 and PLC-γ2 are unique in that they are directly activated by tyrosine phosphorylation. PLC-γ1 generates gradients of diacylglycerol (DAG), which locally activate protein kinase C-α (PKC-α) and guide chemotaxis (Asokan et al., 2014). Co-immunoprecipitation (coIP) assays confirmed the direct binding of both FLNA and PLC-γ1 to PD-L1 (Figure 5E). In addition, proximity ligation assays (PLAs) colocalized PD-L1 and PLC-γ1 at the plasma membrane (Figure 5F). Similarly, we confirmed the colocalization of PD-L1 and both PLC-γ1 and FLNA (Figure 5G). Furthermore, the overexpression of PLC-γ1 enhanced PD-L1-induced migration, whereas siRNAs specific to PLC-γ1, as well as inhibitors of PLC-γ1 and PKC-α, inhibited PD-L1-induced migration (Figure S6K). These lines of evidence are consistent with the possibility that PLC-γ1 relays migration signals downstream to PD-L1.

In addition to cytoskeleton regulation, Rho family GTPases instigate specific transcriptional programs (Treisman et al., 1998). To

(B) Parental PC9ER cells and the G4 clone (KO) were subjected to wound closure assays. Migration fronts are shown at time 0 and 10 h later, and velocities are presented.

(C) Derivatives of PC9ER cells were plated onto fibronectin-coated plates. Thirty minutes later, non-adherent cells were removed and adherent cells were stained (means ± SEMs of triplicates).

(D) Parental PC9ER and PD-L1-KO cells (1×10^4 cells/well) were subjected to ^{3}H-thymidine incorporation assays (averages ± SDs of quadruplicates).

(E) The indicated derivatives of PC9ER cells were incubated for 60 min with bromodeoxyuridine (BrdU), fixed, and subjected to staining for PI and BrdU. Cell-cycle distributions are shown.

(F) Whole extracts of the indicated cells were immunoblotted using the indicated antibodies.

(G and H) Control or KO PC9ER cells were grown in medium containing fetal bovine serum (FBS; 1%) and analyzed for migration as in Figure 3B in the presence of the indicated growth factors.

(I and J) WT and KO derivatives of PC9ER cells (1×10^6) were implanted in NSG (I) or in nu/nu (J) mice (n = 10). Shown are averages ± SDs of tumor volumes.

(K and L) Luciferase-labeled PC9ER (WT) and PD-L1 KO cells (1×10^6) were injected into the tail vein of NSG mice. Ten days later, we determined luminescence signals (1-way ANOVA).

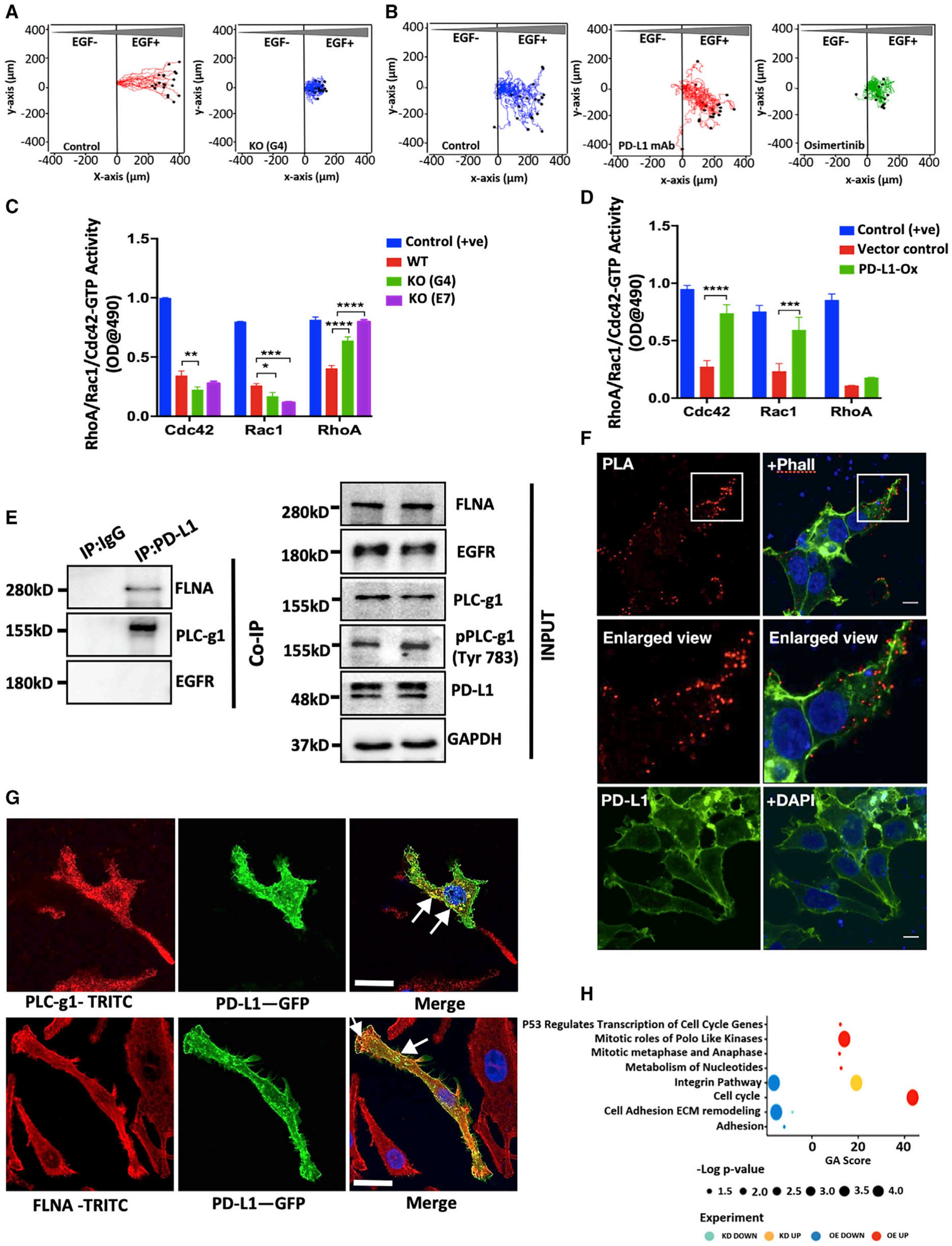

(legend on next page)

uncover the effects induced by PD-L1 and downstream effectors, we isolated RNA from PC9ER cells transiently overexpressing PD-L1, as well as from cells pretreated with PD-L1-specific siRNAs. The differentially expressed genes (Tables S1 and S2) were subjected to enrichment analysis. Enrichment scores of relevant pathways are shown in Figure 5H. Along with multiple regulators of transcription, mitosis, and the cell cycle, many genes controlling cell adhesion were concordantly changed. In summary, we uncovered the direct binding of PD-L1 with both PLC-γ1 and FLNA, as well as unveiled transcriptional programs potentially underlying the proliferative/migratory versus adhesive phenotypes associated with the gain- or loss-of-function of PD-L1, respectively.

Binding with a conserved cytoplasmic motif of PD-L1 enhances stimulatory phosphorylation of PLC-γ1

The short cytoplasmic tail of PD-L1 contains a C-terminal inhibitory sequence and a larger proximal motif that protects cancer cells from IFN cytotoxicity (Gato-Cañas et al., 2017). To map the PLC-γ1 binding site, we deleted the 6 most carboxyl-terminal amino acids of PD-L1 and separately constructed another mutant lacking the conserved 16 terminal amino acids (Figures 6A and 6B). Immunoprecipitation assays indicated that PLC-γ1 binds with the proximal rather than with the terminal motif (Figure 6C). A previous study showed that PLC-γ1 activation requires intramolecular interactions between phosphorylated tyrosine-783 and the C terminus of the enzyme (Poulin et al., 2005). Accordingly, we observed increased tyrosine-783 phosphorylation in A549 cells (low PD-L1 expressors) only if they were prestimulated with EGF (Figures 6D and 6E). Remarkably, when we gradually overexpressed PD-L1 in EGF-stimulated cells, the phosphor-PLC-γ1 signal progressively elevated, but no phosphorylation was observed in the absence of EGF. Conversely, when we knocked down PD-L1, the phosphorylated form of PLC-γ1 was diminished, despite strong activation of EGFR by EGF (Figure S7A). These observations proposed that the PLC-γ1::PD-L1 complex can enhance trans-phosphorylation of PLC-γ1 by active EGFRs. To prevent the direct recruitment of PLC-γ1 to auto-phosphorylated EGFRs, we mutated to phenylalanines all 5 EGFR auto-phosphorylation tyrosines, including Y992 and Y1173, which dock PLC-γ1. The mutant, denoted F5-EGFR, was expressed in cells lacking endogenous EGFR. Despite the undetectable phosphorylation of F5-EGFR, this mutant was still able to enhance tyrosine phosphorylation of PLC-γ1 (Figure 6F). As predicted by the model, pPLC-γ1 signals were observed only when EGFR was activated and PD-L1 was overexpressed. Congruent with the model, proximity ligation signals indicative of PLC-γ1::PD-L1 complexes were enhanced when cells were prestimulated with EGF (Figure S7B). In summary, binding with a conserved segment of PD-L1, which is known to protect from IFN cytotoxicity, enables PLC-γ1 to undergo enhanced phosphorylation and catalytic stimulation in EGF-stimulated cells.

By activating PLC-γ1, PD-L1 molecules enhance EGF-induced calcium flux, DAG production, and PKC-α activation

Through the cleavage of phosphatidylinositol 4,5-bisphosphate (PIP2) and the generation of two second messengers, calcium ions and DAG, upstream activators of PLC-γ1 alter the cytoskeleton and stimulate PKC-α. Hence, we assayed calcium, DAG, and PKC-α. Notably, previous work has established that calcium functions as a key regulator of cell migration (Paupe and Prudent, 2018). To measure cytosolic calcium, PD-L1-GFP-overexpressing cells were preincubated with Calcium Orange. Within 2 min after adding EGF, we observed a large burst of free cytosolic calcium, which decayed 4 min later (Figure 7A). No similar signals were observed when control PC9ER or KO cells were examined (Figure 7B). As an alternative test, we applied flow cytometry on cells that were preloaded with the Indo-1 calcium indicator. Once again, PD-L1-overexpressing cells, unlike the KO and control cells, exhibited large EGF-induced changes in the indicative violet:blue light ratio (Figure 7C).

As expected, PD-L1-overexpressing cells displayed higher DAG levels, relative to the control cells, and the levels measured in KO cells were significantly lower, relative to WT cells (Figure 7D). DAG and Ca^{2+} activate PKC-α, and this typically involves the translocation of the kinases to the plasma membrane. As early as 60 s after stimulating PC9ER cells with EGF, we observed the extensive translocation of PKC-α molecules to the membrane, including the leading edge of migrating cells (Figure 7E). This effect was nearly as large as the effect of phorbol myristate acetate (PMA), a direct activator of PKC-α, but lower signals were observed in KO cells (Figures S7C and S7D). These results

Figure 5. By altering GTP loading onto small GTPases and recruiting phospholipase C-γ1, PD-L1 instigates transcriptional programs and chemotaxis

(A) PC9ER and KO cells were mixed with collagen and then cultured for 48 h. The left parts of chemotaxis chambers were filled with control media and the right sides were filled with EGF-containing media (30 ng/mL). Live imaging was performed for 16 h. The respective rose plots were processed using dedicated software.

(B) PC9ER cells were pretreated with durvalumab (0.05 mg/mL) or osimertinib (40 nM) for 24 h. The cells were later assayed as in (A).

(C and D) Activities of RhoA, RAC1, and CDC42 were determined using an ELISA-based kit. Both PD-L1-overexpressing PC9ER cells and KO clones were used, along with a positive control and an EV (2-way ANOVA).

(E) Extracts of PC9ER cells were subjected to a co-immunoprecipitation (coIP) assay using a control antibody or an antibody to PD-L1. Shown are input blots, along with immunoblots probed for PLC-γ1, FLNA, and PD-L1.

(F) PC9ER cells transiently overexpressing PD-L1 and PLC-γ1 were fixed and probed with the indicated antibodies. Thereafter, cells were processed for PLA that used TRITC (red). Counterstaining used DAPI (blue) and phalloidin-fluorescein isothiocyanate (FITC) (green). The squared areas are magnified. Single antibody controls are shown. Scale bar, 10 μm.

(G) PC9ER cells transiently overexpressing PD-L1-GFP were seeded on coverslips (0.5×10^6). Thereafter, cells were fixed and incubated with PLC-γ1- or FLNA-specific antibodies, followed by a TRITC-conjugated antibody. The arrows mark the co-stained areas. Bars, 10 μm.

(H) RNA was isolated from PC9ER cells transiently overexpressing PD-L1 fused to GFP, or from cells that were pretreated for 48 h with siPD-L1. RNA-seq libraries were sequenced at 10 million reads per sample. Up- and downregulated genes were analyzed using GeneAnalytics (https://geneanalytics.genecards.org/).

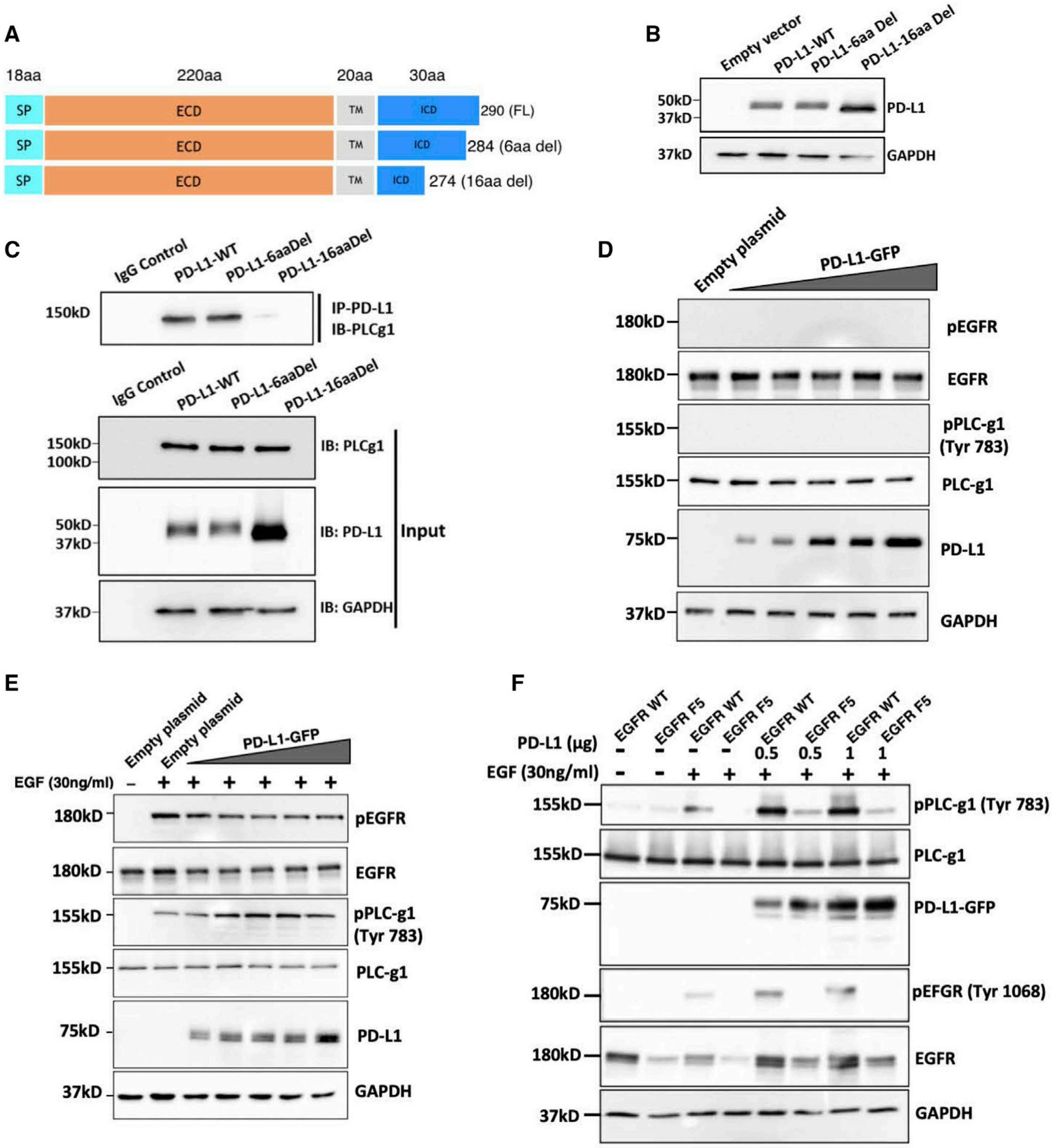

Figure 6. PD-L1 facilitates EGF-induced activation of PLC-γ1, which is recognized by a cytoplasmic motif of PD-L1

(A) Schematic diagrams of full-length PD-L1 and the C-terminally truncated mutants. Full-length PD-L1 comprises a signal peptide (SP), an extracellular domain (ECD), a transmembrane segment (TM) and an intracellular domain (ICD). The numbers of amino acids (aa) in each domain are indicated.

(B) HEK293 cells were pretransfected with plasmids encoding PD-L1 (full length) or the truncation mutants PD-L1-6aaDel or PD-L1-16aaDel. Proteins were extracted 48 h later and resolved using IB.

(C) HEK293 cells were transfected as in (A) and subjected to IP and IB assays. Shown are blots probed for PLC-γ1, PD-L1, and GAPDH.

(D) A549 cells were transfected with increasing amounts of a PD-L1-GFP plasmid (0.5–4 µg). Extracts were resolved using IB.

(E) A549 cells were transfected as in (D). After 48 h, cells were stimulated for 30 min with EGF and analyzed as in (D).

(F) Chinese hamster ovary (CHO) cells were transfected with plasmids encoding EGFR (WT), F5-EGFR (lacking all 5 auto-phosphorylation sites), and PD-L1-GFP (0.5 or 1 µg). Posttransfection, cells were stimulated with EGF for 30 min, and later they were extracted for IB.

established an ability of PD-L1 to regulate intracellular calcium concentrations and stimulate PKC-α. Next, we determined the effects of U73122 (a PLC inhibitor) and PP2 (a blocker of SRC) on RhoA-GTP levels. Notably, previous reports showed that PKC stimulates SRC, which in turn activates p190RhoGAP (Fincham et al., 1999), thereby inhibiting RhoA. In line with a PLC-γ1/PKC/SRC/p190RhoGAP axis, the results presented in Figure 7F showed that both U73122 and PP2 increased RHOA-GTP levels. Along with the other observations we presented, these results offer the following model (see Figure 7G): PD-L1 basally recruits

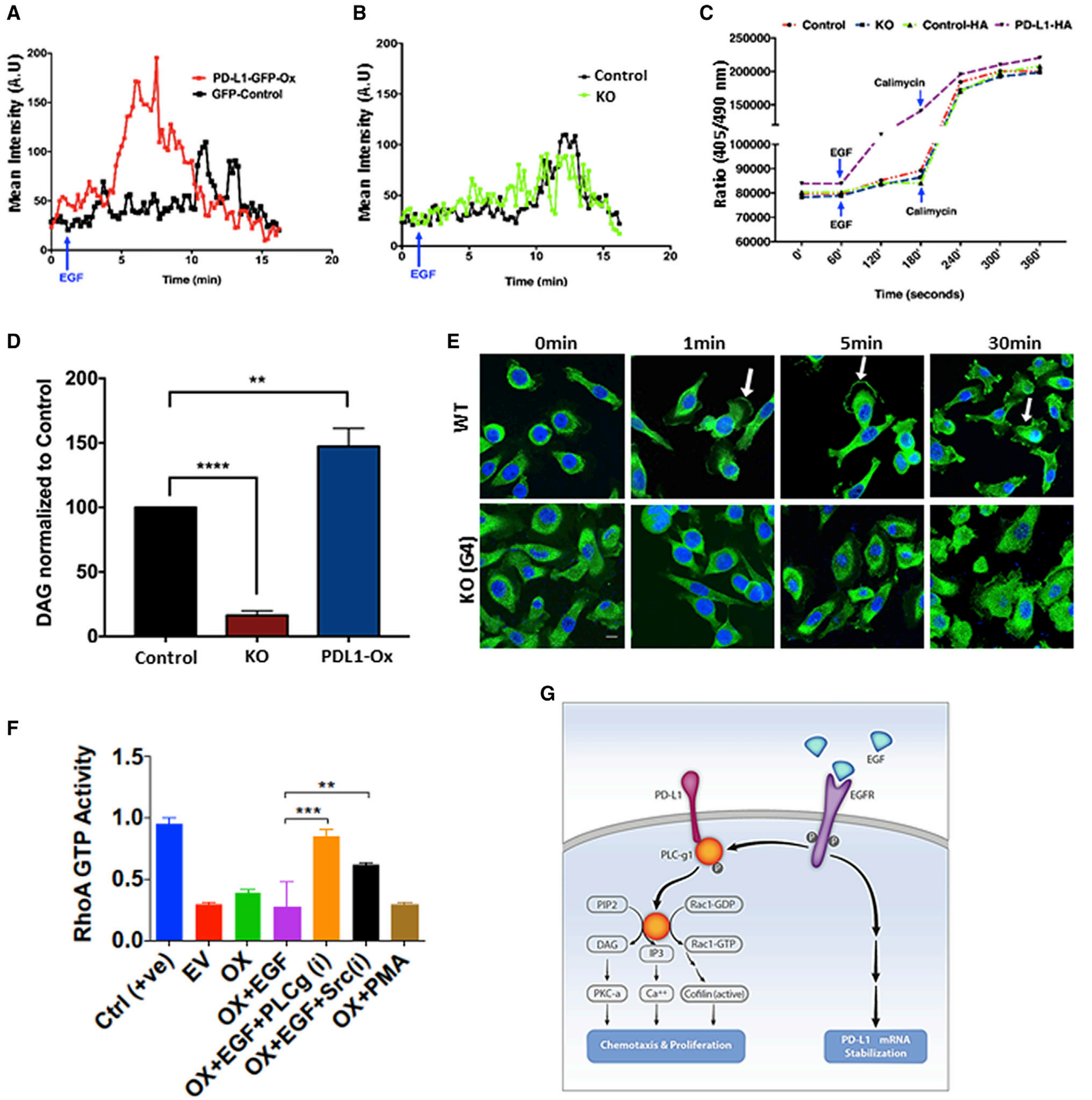

Figure 7. PD-L1 abundance regulates PLC-γ1 phosphorylation, PKC-α activation, and calcium fluxes

(A and B) PC9ER cells were pretransfected with either pGIPZ- EGFP or pGIPZ-PD-L1-EGFP (A). Alternatively, we used PD-L1 KO cells and parental cells (B). Cells were incubated for 30 min with Calcium Orange (2 μM) and stimulated with EGF 2 min after initial recording. The quantification of time-lapse fluorescence was performed over 17 min.

(C) PC9ER, KO, and cells transiently expressing hemagglutinin (HA)-tagged PD-L1 were preloaded with Indo-1. EGF was added 1 min after the beginning of the experiment, and cells were subjected to flow cytometry. Calcimycin (A23187) was added after 180 s (arrows). Ca^{2+} influx was followed by measuring changes in the mean fluorescence ratio of Indo-1 at violet (405 nm) to blue laser (510 nm).

(D) PC9ER cells were transfected with the pGIPZ-EGFP (control) or PD-L1-GFP plasmids. Alternatively, we used PD-L1 KO cells (1.4×10^7). Steady-state DAG levels were measured (4 biological repeats).

(E) Control PC9ER cells and KO cells were serum starved, treated with EGF, and then fixed. Thereafter, cells were incubated with a PKC-α-specific antibody, followed by a secondary, FITC-conjugated antibody. Images were quantified in 2 experiments. Arrows mark PKC-α recruited to the plasma membrane.

(F) The endogenous activity of RhoA was determined. PC9ER cells (0.5×10^6) were transfected with a PD-L1 plasmid (3 μg). After 48 h, cells were serum starved overnight and later preincubated for 60 min with either U73122 (15 μM), a PLC inhibitor, or PP2 (50 μM), a SRC inhibitor. This was followed by stimulation for 30 min at 37°C with either EGF (30 ng/mL) or PMA. Cleared cell extracts were assayed for RhoA GTPase levels. Statistical analyses were performed using 1-way ANOVA.

(legend continued on next page)

inactive PLC-γ1 molecules from the cytosol. Following trans-phosphorylation by mutation- or ligand-activated EGFR molecules, PLC-γ1 shifts to an active state. This initiates an invasive phenotype and loosens matrix adhesion. Notably, no activation of PLC-γ1 occurred when EGFR remained inactive or when PD-L1 abundance was low. This two-hit model of PLC-γ1 activation explains why IFN-γ cannot stimulate cell migration, although it elevates PD-L1 levels, and likewise, EGF only weakly stimulates migration of lung cells devoid of PD-L1.

In summary, along with involvement in immunosuppression, PD-L1 likely evolved as a molecular amplifier of the migratory signals of EGFR. By directly interacting with PLC-γ1, PD-L1 captures for EGFR a major hub able to integrate phosphoinositol signaling, cytosolic calcium, and cytoskeleton reorganization. Conceivably, EGFR-mediated stabilization of the mRNA of PD-L1, together with EGFR- and PD-L1-dependent activation of PLC-γ1, make up a positive feedback loop able to facilitate the oncogenic function of EGFR. In the short term, this translates to the remodeling of the actin cytoskeleton, loosening focal adhesion sites and projecting filopodia, while late-onset transcriptional events establish a motile and invasive phenotype, thought to contribute to immune escape through multiple routes (Terry et al., 2017).

DISCUSSION

While it is clear that growth factors and receptor tyrosine kinases are widely involved in tumor progression (Minn et al., 2005; Witsch et al., 2010), their relevance to the immunosuppressive bed of advanced tumors remains unclear. This is due in part to the multiplicity of stromal factors. The situation is simpler in lung cancer, since genetic manipulations of embryos assigned key roles in the lung epithelium to EGFR ligands (Sibilia and Wagner, 1995). To circumvent the ligand multiplicity issue, we analyzed in patients with lung cancer the transcriptional outcomes of 10 major signaling pathways and correlated them to the level of PD-L1. The results identified the EGFR pathway as the primary non-immunological inducer of PD-L1. This observation is reminiscent of reports that found an association between mutant EGFRs and high PD-L1 (Akbay et al., 2013; Azuma et al., 2014), although exceptions may exist (Soo et al., 2018). It is interesting comparing the induction of PD-L1 by EGF, which is rapid, involves RNA-binding proteins, and leads to EMT, unlike the induction by IFN-γ, which associates with no invasive phenotype. These differences, along with the finding that EGFR trans-phosphorylates PLC-γ1 molecules bound to the cytosolic tail of PD-L1, may relate to the allele-specific resistance of $EGFR^{+}$ tumors to ICBs.

Although short and lacking enzymatic activity, the cytoplasmic domain of PD-L1 mediates a plethora of cellular functions. Furthermore, posttranslational modifications play important roles in regulating the stability and trafficking of PD-L1 (Hsu et al., 2018). Several signaling proteins physically bind with the tail to relay biochemical signals (Escors et al., 2018), including protection from IFN cytotoxicity (Gato-Cañas et al., 2017). By using a genetic screen, we found that PLC-γ1 and filamins serve as components of the PD-L1 signalosome. Of relevance, PLC-γ1 generates DAG, such that a gradient of DAG molecules can locally activate PKC-α (Asokan et al., 2014). In addition, PLC-γ1 generates another second messenger, inositol 1,4,5-trisphosphate (IP3), which elevates cytosolic calcium ions and controls actin-myosin contractions (Paupe and Prudent, 2018). An additional pivotal function of PLC-γ1 entails complex formation with and activation of RAC1 (Li et al., 2009).

Another relevant aspect of PLC-γ1 and PD-L1 relates to AKT. AKT activation enhances the ability of PLC-γ1 to increase cellular motility (Wang et al., 2006). Specifically, phosphorylation by EGFR causes a conformational change of PLC-γ1, thereby allowing interactions with the proline-rich motifs of AKT and phosphorylation of the S1248 of PLC-γ1, which enhances the motility of EGF-stimulated cells. Because several lines of evidence implicate specific PLC isoforms in cellular motility (Wells et al., 2011) and PLC-γ1 plays an important role in actin reorganization (Millarte et al., 2015), the uncovered coupling of PD-L1 to PLC-γ1 explains the strong effects of PD-L1 on EMT, TEM, and metastasis. These effects are mediated by a large protein complex that includes both PLC-γ1 and filamins and docks at membrane-localized PD-L1 molecules. Importantly, complex formation and constant delivery of new PD-L1 molecules to the plasma membrane of EGFR-stimulated cells appear to negate the action of anti-PD-L1 antibodies, thereby diminishing responses to ICBs. However, whether specific alleles of EGFR differentially couple to the PD-L1/PLC-γ1 complex remains to be investigated.

STAR★METHODS

Detailed methods are provided in the online version of this paper and include the following:

- KEY RESOURCES TABLE
- RESOURCE AVAILABILITY
  - Lead contact
  - Materials availability
  - Data and code availability
- EXPERIMENTAL MODEL AND SUBJECT DETAILS
  - Cell lines
  - Mice
- METHOD DETAILS
  - Analysis of patient tumor data
  - Cell cycle analysis
  - TEM and migration assays
  - Cell lysis, immunoblotting, and co-immunoprecipitation assays
  - RNA isolation and real-time PCR analysis
  - Chemotaxis assays

(G) The model depicts the herein reported interactions between a short cytoplasmic segment of PD-L1 and PLC-γ1. Formation of this complex enhances the activation of PLC-γ1 by ligand- or mutation-activated EGFRs. Once stimulated by means of phosphorylation, PLC-γ1 degrades phosphatidylinositol 4,5-bisphosphate (PIP2) to generate both diacylglycerol (which activates PKC) and inositol triphosphate (which elevates cytoplasmic Ca^{2+}). The direct interaction between PD-L1 and PLC-γ1 may explain the herein reported involvement of PD-L1 in chemotaxis and metastasis.

- ○ *In vitro* knockout of the gene encoding for PD-L1
- ○ Proximity ligation assays (PLA)
- ○ Intracellular calcium measurements
- ○ Lung metastasis assays
- QUANTIFICATION AND STATISTICAL ANALYSIS

SUPPLEMENTAL INFORMATION

Supplemental information can be found online at https://doi.org/10.1016/j.celrep.2021.109181.

ACKNOWLEDGMENTS

We thank Tomer Meir Salame for experimental help and Julian Downwards for sharing plasmids. Y.Y. is the incumbent of the Harold and Zelda Goldenberg Professorial Chair in Molecular Cell Biology. Our studies have been supported by the Israel Science Foundation (ISF), the Israel Cancer Research Fund (ICRF), the European Research Council (ERC), and the Dr. Miriam and Sheldon G. Adelson Medical Research Foundation.

AUTHOR CONTRIBUTIONS

S.G. and Y.Y. designed the experiments and wrote the manuscript. N.B.N., A.N., S.P., A.S., S.M., S.W.-K., M.L., D.D.G., J.G., L.K., G.E., A.B., and Y.L. performed the experiments. S.P., Y.L., and E.R. performed the bioinformatics analyses. H.G.-H., S.W., M.O., M.L., B.G., and E.R. reviewed the manuscript.

DECLARATION OF INTERESTS

The authors declare no competing interests.

Received: April 27, 2020
Revised: October 20, 2020
Accepted: May 4, 2021
Published: May 25, 2021

REFERENCES

Akbay, E.A., Koyama, S., Carretero, J., Altabef, A., Tchaicha, J.H., Christensen, C.L., Mikse, O.R., Cherniack, A.D., Beauchamp, E.M., Pugh, T.J., et al. (2013). Activation of the PD-1 pathway contributes to immune escape in EGFR-driven lung tumors. Cancer Discov. *3*, 1355–1363.

Akinleye, A., and Rasool, Z. (2019). Immune checkpoint inhibitors of PD-L1 as cancer therapeutics. J. Hematol. Oncol. *12*, 92.

Asokan, S.B., Johnson, H.E., Rahman, A., King, S.J., Rotty, J.D., Lebedeva, I.P., Haugh, J.M., and Bear, J.E. (2014). Mesenchymal chemotaxis requires selective inactivation of myosin II at the leading edge via a noncanonical PLCγ/PKCα pathway. Dev. Cell *31*, 747–760.

Azuma, K., Ota, K., Kawahara, A., Hattori, S., Iwama, E., Harada, T., Matsumoto, K., Takayama, K., Takamori, S., Kage, M., et al. (2014). Association of PD-L1 overexpression with activating EGFR mutations in surgically resected nonsmall-cell lung cancer. Ann. Oncol. *25*, 1935–1940.

Chen, L., Gibbons, D.L., Goswami, S., Cortez, M.A., Ahn, Y.H., Byers, L.A., Zhang, X., Yi, X., Dwyer, D., Lin, W., et al. (2014). Metastasis is regulated via microRNA-200/ZEB1 axis control of tumour cell PD-L1 expression and intratumoral immunosuppression. Nat. Commun. *5*, 5241.

Chen, N., Fang, W., Zhan, J., Hong, S., Tang, Y., Kang, S., Zhang, Y., He, X., Zhou, T., Qin, T., et al. (2015). Upregulation of PD-L1 by EGFR Activation Mediates the Immune Escape in EGFR-Driven NSCLC: Implication for Optional Immune Targeted Therapy for NSCLC Patients with EGFR Mutation. J. Thorac. Oncol. *10*, 910–923.

Chen, L., Xiong, Y., Li, J., Zheng, X., Zhou, Q., Turner, A., Wu, C., Lu, B., and Jiang, J. (2017). PD-L1 Expression Promotes Epithelial to Mesenchymal Transition in Human Esophageal Cancer. Cell. Physiol. Biochem. *42*, 2267–2280.

Clark, C.A., Gupta, H.B., Sareddy, G., Pandeswara, S., Lao, S., Yuan, B., Drerup, J.M., Padron, A., Conejo-Garcia, J., Murthy, K., et al. (2016). Tumor-Intrinsic PD-L1 Signals Regulate Cell Growth, Pathogenesis, and Autophagy in Ovarian Cancer and Melanoma. Cancer Res. *76*, 6964–6974.

Coelho, M.A., de Carné Trécesson, S., Rana, S., Zecchin, D., Moore, C., Molina-Arcas, M., East, P., Spencer-Dene, B., Nye, E., Barnouin, K., et al. (2017). Oncogenic RAS Signaling Promotes Tumor Immunoresistance by Stabilizing PD-L1 mRNA. Immunity *47*, 1083–1099.e6.

Debnath, J., and Brugge, J.S. (2005). Modelling glandular epithelial cancers in three-dimensional cultures. Nat. Rev. Cancer *5*, 675–688.

Dong, H., Strome, S.E., Salomao, D.R., Tamura, H., Hirano, F., Flies, D.B., Roche, P.C., Lu, J., Zhu, G., Tamada, K., et al. (2002). Tumor-associated B7-H1 promotes T-cell apoptosis: a potential mechanism of immune evasion. Nat. Med. *8*, 793–800.

Dong, P., Xiong, Y., Yu, J., Chen, L., Tao, T., Yi, S., Hanley, S.J.B., Yue, J., Watari, H., and Sakuragi, N. (2018). Control of PD-L1 expression by miR-140/142/340/383 and oncogenic activation of the OCT4-miR-18a pathway in cervical cancer. Oncogene *37*, 5257–5268.

Escors, D., Gato-Cañas, M., Zuazo, M., Arasanz, H., García-Granda, M.J., Vera, R., and Kochan, G. (2018). The intracellular signalosome of PD-L1 in cancer cells. Signal Transduct. Target. Ther. *3*, 26.

Etienne-Manneville, S., and Hall, A. (2003). Cdc42 regulates GSK-3beta and adenomatous polyposis coli to control cell polarity. Nature *421*, 753–756.

Evans, M., O'Sullivan, B., Hughes, F., Mullis, T., Smith, M., Trim, N., and Taniere, P. (2020). The Clinicopathological and Molecular Associations of PD-L1 Expression in Non-small Cell Lung Cancer: Analysis of a Series of 10,005 Cases Tested with the 22C3 Assay. Pathol. Oncol. Res. *26*, 79–89.

Fincham, V.J., Chudleigh, A., and Frame, M.C. (1999). Regulation of p190 Rho-GAP by v-Src is linked to cytoskeletal disruption during transformation. J. Cell Sci. *112*, 947–956.

Fromont-Racine, M., Rain, J.C., and Legrain, P. (1997). Toward a functional analysis of the yeast genome through exhaustive two-hybrid screens. Nat. Genet. *16*, 277–282.

Gainor, J.F., Shaw, A.T., Sequist, L.V., Fu, X., Azzoli, C.G., Piotrowska, Z., Huynh, T.G., Zhao, L., Fulton, L., Schultz, K.R., et al. (2016). EGFR Mutations and ALK Rearrangements Are Associated with Low Response Rates to PD-1 Pathway Blockade in Non-Small Cell Lung Cancer: A Retrospective Analysis. Clin. Cancer Res. *22*, 4585–4593.

Garcia-Diaz, A., Shin, D.S., Moreno, B.H., Saco, J., Escuin-Ordinas, H., Rodriguez, G.A., Zaretsky, J.M., Sun, L., Hugo, W., Wang, X., et al. (2017). Interferon Receptor Signaling Pathways Regulating PD-L1 and PD-L2 Expression. Cell Rep. *19*, 1189–1201.

Gato-Cañas, M., Zuazo, M., Arasanz, H., Ibañez-Vea, M., Lorenzo, L., Fernandez-Hinojal, G., Vera, R., Smerdou, C., Martisova, E., Arozarena, I., et al. (2017). PDL1 Signals through Conserved Sequence Motifs to Overcome Interferon-Mediated Cytotoxicity. Cell Rep. *20*, 1818–1829.

Hastings, K., Yu, H.A., Wei, W., Sanchez-Vega, F., DeVeaux, M., Choi, J., Rizvi, H., Lisberg, A., Truini, A., Lydon, C.A., et al. (2019). EGFR mutation subtypes and response to immune checkpoint blockade treatment in non-small-cell lung cancer. Ann. Oncol. *30*, 1311–1320.

Hellmann, M.D., Nathanson, T., Rizvi, H., Creelan, B.C., Sanchez-Vega, F., Ahuja, A., Ni, A., Novik, J.B., Mangarin, L.M.B., Abu-Akeel, M., et al. (2018). Genomic Features of Response to Combination Immunotherapy in Patients with Advanced Non-Small-Cell Lung Cancer. Cancer Cell *33*, 843–852.e4.

Hsu, J.M., Li, C.W., Lai, Y.J., and Hung, M.C. (2018). Posttranslational Modifications of PD-L1 and Their Applications in Cancer Therapy. Cancer Res. *78*, 6349–6353.

Huttlin, E.L., Ting, L., Bruckner, R.J., Gebreab, F., Gygi, M.P., Szpyt, J., Tam, S., Zarraga, G., Colby, G., Baltier, K., et al. (2015). The BioPlex Network: A Systematic Exploration of the Human Interactome. Cell *162*, 425–440.

Irie, H.Y., Pearline, R.V., Grueneberg, D., Hsia, M., Ravichandran, P., Kothari, N., Natesan, S., and Brugge, J.S. (2005). Distinct roles of Akt1 and Akt2 in

regulating cell migration and epithelial-mesenchymal transition. J. Cell Biol. *171*, 1023–1034.

Kim, S., Koh, J., Kim, M.-Y., Kwon, D., Go, H., Kim, Y.A., Jeon, Y.K., and Chung, D.H. (2016). PD-L1 expression is associated with epithelial-to-mesenchymal transition in adenocarcinoma of the lung. Hum. Pathol. *58*, 7–14.

Lee, C.K., Man, J., Lord, S., Cooper, W., Links, M., Gebski, V., Herbst, R.S., Gralla, R.J., Mok, T., and Yang, J.C. (2018). Clinical and Molecular Characteristics Associated With Survival Among Patients Treated With Checkpoint Inhibitors for Advanced Non-Small Cell Lung Carcinoma: A Systematic Review and Meta-analysis. JAMA Oncol. *4*, 210–216.

Li, S., Wang, Q., Wang, Y., Chen, X., and Wang, Z. (2009). PLC-gamma1 and Rac1 coregulate EGF-induced cytoskeleton remodeling and cell migration. Mol. Endocrinol. *23*, 901–913.

Li, C.W., Lim, S.O., Xia, W., Lee, H.H., Chan, L.C., Kuo, C.W., Khoo, K.H., Chang, S.S., Cha, J.H., Kim, T., et al. (2016). Glycosylation and stabilization of programmed death ligand-1 suppresses T-cell activity. Nat. Commun. *7*, 12632.

Li, J., Chen, L., Xiong, Y., Zheng, X., Xie, Q., Zhou, Q., Shi, L., Wu, C., Jiang, J., and Wang, H. (2017). Knockdown of PD-L1 in Human Gastric Cancer Cells Inhibits Tumor Progression and Improves the Cytotoxic Sensitivity to CIK Therapy. Cell. Physiol. Biochem. *41*, 907–920.

Lou, Y., Diao, L., Cuentas, E.R., Denning, W.L., Chen, L., Fan, Y.H., Byers, L.A., Wang, J., Papadimitrakopoulou, V.A., Behrens, C., et al. (2016). Epithelial-Mesenchymal Transition Is Associated with a Distinct Tumor Microenvironment Including Elevation of Inflammatory Signals and Multiple Immune Checkpoints in Lung Adenocarcinoma. Clin. Cancer Res. *22*, 3630–3642.

Millarte, V., Boncompain, G., Tillmann, K., Perez, F., Sztul, E., and Farhan, H. (2015). Phospholipase C γ1 regulates early secretory trafficking and cell migration via interaction with p115. Mol. Biol. Cell *26*, 2263–2278.

Minn, A.J., Gupta, G.P., Siegel, P.M., Bos, P.D., Shu, W., Giri, D.D., Viale, A., Olshen, A.B., Gerald, W.L., and Massagué, J. (2005). Genes that mediate breast cancer metastasis to lung. Nature *436*, 518–524.

Nair, N.U., Das, A., Rogkoti, V.-M., Fokkelman, M., Marcotte, R., de Jong, C.G., Koedoot, E., Lee, J.S., Meilijson, I., Hannenhalli, S., et al. (2019). Migration rather than proliferation transcriptomic signatures are strongly associated with breast cancer patient survival. Sci. Rep. *9*, 10989.

Nobes, C.D., and Hall, A. (1999). Rho GTPases control polarity, protrusion, and adhesion during cell movement. J. Cell Biol. *144*, 1235–1244.

Ota, K., Azuma, K., Kawahara, A., Hattori, S., Iwama, E., Tanizaki, J., Harada, T., Matsumoto, K., Takayama, K., Takamori, S., et al. (2015). Induction of PD-L1 Expression by the EML4-ALK Oncoprotein and Downstream Signaling Pathways in Non-Small Cell Lung Cancer. Clin. Cancer Res. *21*, 4014–4021.

Pankov, R., Endo, Y., Even-Ram, S., Araki, M., Clark, K., Cukierman, E., Matsumoto, K., and Yamada, K.M. (2005). A Rac switch regulates random versus directionally persistent cell migration. J. Cell Biol. *170*, 793–802.

Parsa, A.T., Waldron, J.S., Panner, A., Crane, C.A., Parney, I.F., Barry, J.J., Cachola, K.E., Murray, J.C., Tihan, T., Jensen, M.C., et al. (2007). Loss of tumor suppressor PTEN function increases B7-H1 expression and immunoresistance in glioma. Nat. Med. *13*, 84–88.

Paupe, V., and Prudent, J. (2018). New insights into the role of mitochondrial calcium homeostasis in cell migration. Biochem. Biophys. Res. Commun. *500*, 75–86.

Pegtel, D.M., Ellenbroek, S.I., Mertens, A.E., van der Kammen, R.A., de Rooij, J., and Collard, J.G. (2007). The Par-Tiam1 complex controls persistent migration by stabilizing microtubule-dependent front-rear polarity. Curr. Biol. *17*, 1623–1634.

Petrie, R.J., Doyle, A.D., and Yamada, K.M. (2009). Random versus directionally persistent cell migration. Nat. Rev. Mol. Cell Biol. *10*, 538–549.

Poulin, B., Sekiya, F., and Rhee, S.G. (2005). Intramolecular interaction between phosphorylated tyrosine-783 and the C-terminal Src homology 2 domain activates phospholipase C-gamma1. Proc. Natl. Acad. Sci. USA *102*, 4276–4281.

Rajagopalan, L.E., Burkholder, J.K., Turner, J., Culp, J., Yang, N.S., and Malter, J.S. (1995). Granulocyte-macrophage colony-stimulating factor mRNA stabilization enhances transgenic expression in normal cells and tissues. Blood *86*, 2551–2558.

Ran, F.A., Hsu, P.D., Wright, J., Agarwala, V., Scott, D.A., and Zhang, F. (2013). Genome engineering using the CRISPR-Cas9 system. Nat. Protoc. *8*, 2281–2308.

Rizvi, N.A., Hellmann, M.D., Snyder, A., Kvistborg, P., Makarov, V., Havel, J.J., Lee, W., Yuan, J., Wong, P., Ho, T.S., et al. (2015). Cancer immunology. Mutational landscape determines sensitivity to PD-1 blockade in non-small cell lung cancer. Science *348*, 124–128.

Rizvi, H., Sanchez-Vega, F., La, K., Chatila, W., Jonsson, P., Halpenny, D., Plodkowski, A., Long, N., Sauter, J.L., Rekhtman, N., et al. (2018). Molecular Determinants of Response to Anti-Programmed Cell Death (PD)-1 and Anti-Programmed Death-Ligand 1 (PD-L1) Blockade in Patients With Non-Small-Cell Lung Cancer Profiled With Targeted Next-Generation Sequencing. J. Clin. Oncol. *36*, 633–641.

Shao, Q.-Q., Zhang, T.-P., Zhao, W.-J., Liu, Z.W., You, L., Zhou, L., Guo, J.C., and Zhao, Y.P. (2016). Filamin A: Insights into Its Exact Role in Cancers. Pathol. Oncol. Res. *22*, 245–252.

Sharma, P., and Allison, J.P. (2015). The future of immune checkpoint therapy. Science *348*, 56–61.

Sibilia, M., and Wagner, E.F. (1995). Strain-dependent epithelial defects in mice lacking the EGF receptor. Science *269*, 234–238.

Soo, R.A., Lim, S.M., Syn, N.L., Teng, R., Soong, R., Mok, T.S.K., and Cho, B.C. (2018). Immune checkpoint inhibitors in epidermal growth factor receptor mutant non-small cell lung cancer: current controversies and future directions. Lung Cancer *115*, 12–20.

Terry, S., Savagner, P., Ortiz-Cuaran, S., Mahjoubi, L., Saintigny, P., Thiery, J.P., and Chouaib, S. (2017). New insights into the role of EMT in tumor immune escape. Mol. Oncol. *11*, 824–846.

Topalian, S.L., Drake, C.G., and Pardoll, D.M. (2015). Immune checkpoint blockade: a common denominator approach to cancer therapy. Cancer Cell *27*, 450–461.

Treisman, R., Alberts, A.S., and Sahai, E. (1998). Regulation of SRF activity by Rho family GTPases. Cold Spring Harb. Symp. Quant. Biol. *63*, 643–651.

Tu, X., Qin, B., Zhang, Y., Zhang, C., Kahila, M., Nowsheen, S., Yin, P., Yuan, J., Pei, H., Li, H., et al. (2019). PD-L1 (B7-H1) Competes with the RNA Exosome to Regulate the DNA Damage Response and Can Be Targeted to Sensitize to Radiation or Chemotherapy. Mol. Cell *74*, 1215–1226.e4.

Wang, Y., Wu, J., and Wang, Z. (2006). Akt binds to and phosphorylates phospholipase C-gamma1 in response to epidermal growth factor. Mol. Biol. Cell *17*, 2267–2277.

Wang, Y., Hu, J., Wang, Y., Ye, W., Zhang, X., Ju, H., Xu, D., Liu, L., Ye, D., Zhang, L., et al. (2018). EGFR activation induced Snail-dependent EMT and myc-dependent PD-L1 in human salivary adenoid cystic carcinoma cells. Cell Cycle *17*, 1457–1470.

Wells, A., Chao, Y.L., Grahovac, J., Wu, Q., and Lauffenburger, D.A. (2011). Epithelial and mesenchymal phenotypic switchings modulate cell motility in metastasis. Front. Biosci. *16*, 815–837.

Witsch, E., Sela, M., and Yarden, Y. (2010). Roles for growth factors in cancer progression. Physiology (Bethesda) *25*, 85–101.

Yamada, T., Hirai, S., Katayama, Y., Yoshimura, A., Shiotsu, S., Watanabe, S., Kikuchi, T., Hirose, K., Kubota, Y., Chihara, Y., et al. (2019). Retrospective efficacy analysis of immune checkpoint inhibitors in patients with EGFR-mutated non-small cell lung cancer. Cancer Med. *8*, 1521–1529.

Zhang, W., Pang, Q., Yan, C., Wang, Q., Yang, J., Yu, S., Liu, X., Yuan, Z., Wang, P., and Xiao, Z. (2017). Induction of PD-L1 expression by epidermal growth factor receptor-mediated signaling in esophageal squamous cell carcinoma. OncoTargets Ther. *10*, 763–771.

Zhong, F., Cheng, X., Sun, S., and Zhou, J. (2017). Transcriptional activation of PD-L1 by Sox2 contributes to the proliferation of hepatocellular carcinoma cells. Oncol. Rep. *37*, 3061–3067.

STAR★METHODS

KEY RESOURCES TABLE

REAGENT or RESOURCE	SOURCE	IDENTIFIER
Antibodies		
Rabbit monoclonal anti-PD-L1	Cell Signaling Technology	Cat# 13684S; RRID: AB_2687655
Mouse monoclonal anti-Brdu	Invitrogen	Cat# 175071; RRID:AB_11040534
Mouse monoclonal anti-GAPDH	Merck	Cat# MAB374; RRID:AB_2107445
Mouse monoclonal anti-RAS-GAP	Santa Cruz Biotechnology	Cat# SC63;RRID:AB_628206
Rabbit monoconal anti-pSTAT3	Cell Signaling Technology	Cat# 9145; RRID:AB_2491009
Mouse monoclonal anti-EGFR	Santa Cruz Biotechnology	CAT# SC101; RRID:AB_627494
Rabbit Anti-pEGFR	Cell Signaling Technology	Cat#2234; RRID:AB_331701
Rabbit anti-STAT1	Cell Signaling Technology	Cat # 9172; RRID:AB_2198300
Rabbit monoconal anti-MEK	Cell Signaling Technology	Cat #9146; RRID:AB_2138020
Rabbit anti-AKT	Cell Signaling Technology	Cat#4691; RRID:AB_915783
Rabbit Anti-pAKT	Cell Signaling Technology	Cat#9271; RRID:AB_329825
Rabbit monoclonal IgG Isotype control	Abcam	Cat# Ab172730; RRID:AB_2687931
Goat polyclonal IgG Control	R & D systems	Cat# Ab108C; RRID:AB_354267
Mouse polyclonal IgG control	Abcam	Cat# ab37355; RRID:AB_2665484
Mouse IgG1 anti-paxillin	BD Biosciences	Cat# 610052; RRID:AB_397464
PLC-g1	Cell Signaling Technology	Cat#5690; RRID:AB_10691383
PD-L1-FITC	Biolegend Biosciences	Cat #393605; RRID:AB_2734471
pPLC-g1	Cell Signaling Technology	Cat#14008; RRID:AB_2728690
moue monoclonal anti- STAT3	Cell Signaling Technology	Cat# 9139; RRID:AB_331757
Rabbit monoclonal anti-ERK	Cell Signaling Technology	Cat# 4695; RRID:AB_390779
Rabbit monoclonal anti-pERK	Cell Signaling Technology	Cat# 9101; RRID:AB_331646
p-Tyr Antibody (PY20)	Santa Cruz Biotechnology	Cat# 508; RRID:AB_628122
Tristetraprolin (D1I3T) Rabbit	Cell Signaling Technology	Cat# 71632; RRID:AB_2799806
AUF1/hnRNP D (D6O4F) Rabbit	Cell Signaling Technology	Cat# 12382; RRID:AB_2616009
E-Cadherin (24E10) Rabbit	Cell Signaling Technology	Cat# 3195; RRID:AB_2291471
N-Cadherin (D4R1H) XP® Rabbit	Cell Signaling Technology	Cat# 13116; RRID:AB_2687616
β-Catenin (D10A8) XP® Rabbit	Cell Signaling Technology	Cat# 8480; RRID:AB_11127855
Rabbit polyclonal claudin-1	Abcam	Cat# ab 15098; RRID:AB_301644
Rabbit polyclonal anti-fibronectin antibody	Abcam	Cat# ab 2413; RRID:AB_2262874
Vimentin (D21H3) XP® Rabbit mAb	Cell Signaling Technology	Cat# 5741; RRID:AB_10695459
VASP (9A2) Rabbit mAb	Cell Signaling Technology	Cat# 3132; RRID:AB_2213393
Cofilin (D3F9) XP® Rabbit mAb	Cell Signaling Technology	Cat# 5175; RRID:AB_10622000
α-Actinin (D6F6) XP® Rabbit mAb	Cell Signaling Technology	Cat# 6487; RRID:AB_11179206
Anti-Filamin A Antibody, clone TI10	Millipore Sigma	Cat# MAB1680; RRID:AB_94323
Mouse monoclonal PKC α Antibody (H-7)	Santa Cruz	Cat# 8393; RRID:AB_628142
Tecentriq (atezolizumab injection)	Genentech (gift from Prof Michal, Hadassah medical center)	N/A
Durvalumab	Astrazeneca (gift from Prof Michal, Hadassah medical center)	N/A
Chemicals, peptides, and recombinant proteins		
Liquid scintillation cocktail	Perkin Elmer, USA	Cat# 6013329
d-Luciferin	Perkin Elmer, USA	Cat# 122799
3 [H]-thymidine	Perkin Elmer, USA	Cat# NET027Z001MC

(*Continued on next page*)

Continued

REAGENT or RESOURCE	SOURCE	IDENTIFIER
Phalloidin	Sigma-Aldrich	Cat# P1951; P5282
Fibronectin	Biological Industries	Cat# 05-750-1F
Cyclohexamide	Sigma	Cat# 66-81-8
ActinomycinD	Sigma	Cat# A1410
Selumetinib (AZD6244)	Selleckchem	Cat# S1008
AZD9291	Selleckchem	Cat# S7297
BEZ235	Selleckchem	Cat# S1009
Afatinib	Selleckchem	Cat# S1011
Lapatinib	Selleckchem	Cat# S2111
Erlotinib	Selleckchem	Cat# S1023
EGF	Sigma	Cat# E9644
MTT	Sigma	Cat# M5655
PMA	Sigma	Cat# P1585
U73122	Sigma	Cat# U6756
RO318220	Millipore Sigma	Cat# 557520
DMSO	Sigma	Cat# D2438
Recombinant Human IFN-γ	Peprotech	Cat# 300-02
TGF-b	Peprotech	Cat# 100-21100-21
Picro-sirius red solution	Abcam	Cat# Ab246832
FGF-7	Peprotech	Cat# 100-18C
Recombinant Human Amphiregulin	Peprotech	Cat# 100-55B
NGF	Peprotech	Cat # 450-01
HGF	Peprotech	Cat# 100-39H
Calcimycin	Abcam	Cat# A23187
**Critical commercial assays**		
Transwell	COSTAR	Cat# 3422
Matrigel Invasion Chamber	BD Biosciences	Cat# FAL354480
Genomic Isolation Kit	Invitrogen	Cat# K182001
Midi Prep Kit	Qiagen	Cat# 12143
SYBR Green PCR Master Mix	Thermo Fisher Scientific	Cat# 4309155
25 Culture-Inserts 2 Well for self-insertion	Ibidi	Cat# 80209
High-Capacity cDNA Reverse Transcription Kit	Thermo Fisher Scientific	Cat# 4368814
Dual-Luciferase Reporter Assay System	Promega	Cat# E1910
VEGF elisa Kit	R & D systems	Cat# CY293B
Duolink *In Situ* Red Starter Kit Mouse/ Rabbit	Millipore Sigma	Cat# DUO92101
Lipofectamine LTX and Plus	Invitrogen	Cat# 15338100
Rho/Rac/cdc42 G-LISA activation kit	Cytoskeleton, Inc	Cat# BK135
3D assay kit	Cultrex	Cat# 3500096K
FluoroBlok	Corning	Cat# 351157
Luciferase kit	Promega	Cat# E1910
RNA Isolation kit	QIAGEN	Cat# 2302350
**Deposited data**		
RNA sequencing data	This paper	GEO (https://www.ncbi.nlm.nih.gov/geo/query/acc.cgi) The accession code is GSE 171650.

(*Continued on next page*)

Continued

REAGENT or RESOURCE	SOURCE	IDENTIFIER
Experimental models: Cell lines		
Human: MDA-MB-231	ATCC	Cat# HTB-26; RRID:CVCL_0062
Human: HEK293T	ATCC	Cat# CRL-3216; RRID:CVCL_0063
MCF10A	ATCC	Cat# CRL-3216; RRID:CVCL_0063
HUVEC	ATCC	N/A
PC9ER	Gift from Julian Downward, Francis Crick Institute, London	N/A
PC9	ATCC	N/A
H1975	ATCC	N/A
H1299	ATCC	Cat# CRL-5803; RRID:CVCL_0060
A549	ATCC	Cat# CCL-185; RRID:CVCL_0023
Experimental models: Organisms/strains		
NOG	Jackson	Cat# HTB-22
NUDE	Jackson	Cat#2019
Oligonucleotides		
siPD-L1	GE healthcare	R-015836-00-0005
siPD-L1	Dharmacon/ Horizon discovery	L-015836-01-0005
siPLCg-1	Dharmacon/ Horizon discovery	L-003559-00-0010
siEGFR	Dharmacon/ Horizon discovery	L-003114-00-0010
Si-STAT3	Dharmacon/ Horizon discovery	L-003544-00-0010
siMEK1/2	Dharmacon/ Horizon discovery	L-003301-00-0010
siAKT	Dharmacon/ Horizon discovery	L-003000-00-0010
Recombinant DNA		
Sh-PD-L1 – SMARTvector Lentiviral shRNA	Dharmacon/ Horizon discovery	V3SH7596-00EG29126
PGL3 basic	Promega	Cat# E1751
pGL3 3′UTR reporter MUT 1.3 kb CD274 Hs 3′UTR	Addgene	Cat# 107010; RRID:Addgene_107010
pcDNA3-EGFP	Addgene	Cat# 13031; RRID:Addgene_13031
pGIPZ-PD-L1-EGFP	Addgene	Cat# 120933; RRID:Addgene_120933
PLCG-1 plasmid full length	Gift for Prof Hanagan, King college London	N/A
pGL3 3′UTR reporter WT 1.3 kb CD274	Addgene	Cat# 107009; RRID:Addgene_107009
PD-L1- HA plasmid	Gift for Prof Mien chi huang, MDACC	N/A
PD-L1 6 amino acid deletion	This paper	N/A
PD-L1 16 amino acid deletion	This paper	N/A
Software and algorithms		
ImageJ	National Institutes of Health, Bethesda, USA	https://imagej.nih.gov/ij/
Prism 8	Graph pad	https://www.graphpad.com/scientific-software/prism/
BD FACS Diva software v8.0.1	BD Biosciences	https://www.zmbh.uni-heidelberg.de/Central_Services/Imaging_Facility/info/780ZEN2010.pdf
LSM 880 Zeiss	Zeiss	https://www.bdbiosciences.com/en-us/instruments/research-instruments/research-software/flow-cytometry-acquisition/facsdiva-software
IVIS	Perkin Elmer, USA	https://www.perkinelmer.com/lab-products-and-services/resources/in-vivo-imaging-software-downloads.html#LivingImage

(*Continued on next page*)

Continued

REAGENT or RESOURCE	SOURCE	IDENTIFIER
Primers		
Human CD274	**Forward**: TATGGTGGTGCCGACTACAA	N/A
Human CD274	**Reverse**: TGGCTCCCAGAATTACCAAG	N/A
Human Actin	**Forward**: CACCAACTGGGACGACAT	N/A
Human Actin	**Reverse**: ACAGCCTGGATAGCAACG	N/A
Human Zeb1	**Forward**: GTGGCGGTAGATGGTAAT	N/A
Human Zeb1	**Reverse**: CTGTTTGTAGCGACTGGA	N/A
Human Twist	**Forward**: CGGGAGTCCGCAGTCTTA	N/A
Human Twist	**Reverse**:GCTTGAGGGTCTGAATCTTG	N/A
Human Vimentin	**Forward**: CCAGGCAAAGCAGGAGTC	N/A
Human Vimentin	**Reverse**: CGAAGGTGACGAGCCATT	N/A
Human Snail	**Forward**: CCCCAATCGGAAGCCTAA	N/A
Human Snail	**Reverse**: CCTTTCCCACTGTCCTCAT	N/A
Human E-Cadh	**Forward**: CTGAGAACGAGGCTAACG	N/A
Human E-Cadh	**Reverse**: TTCACATCCAGCACATCC	N/A
Human N-Cadh	**Forward**:GGTGGAGGAGAAGAAGACCAG	N/A
Human N-Cadh	**Reverse**: GGCATCAGGCTCCACAGT	N/A

RESOURCE AVAILABILITY

Lead contact

Further information and requests for reagents should be directed to Yosef Yarden (yosef.yarden@weizmann.ac.il).

Materials availability

All materials we generated will be made available upon request. The sources and identifiers of all materials are listed in the Key resources table.

Data and code availability

The RNA sequencing data generated during this study are available at GEO (https://www.ncbi.nlm.nih.gov/geo/query/acc.cgi) The accession code is GSE 171650.

EXPERIMENTAL MODEL AND SUBJECT DETAILS

Cell lines

Erlotinib-resistant PC9ER cells (EGFR del746–750 + T790M) are *in vitro* derivatives of the PC9 (del746–750) cell line. The H1975 lung cancer cell line (EGFR mutations L858R and T790M) was obtained from the American Type Tissue Culture Collection (ATCC). All lung cancer cell lines were maintained in RPMI 1640 supplemented with 10% fetal calf serum (FCS) and antibiotics. NL20, an immortalized human lung epithelial cell line, were maintained in F12K medium. MCF10A, non-malignant breast epithelial cells, obtained from ATCC and were maintained in Dulbecco's modified Eagle (DMEM)/F12 medium supplemented with 5% horse serum, insulin, cholera toxin and hydrocortisone.

Mice

All animal studies were approved by the Weizmann Institute's Review Board (IRB) and adhered to the NIH Guide for the Care and Use of Laboratory Animals. CD1 nu/nu mice (female, 5-6 weeks old) were injected subcutaneously with cancer cells (3-4x10^6 per mouse). Antibodies were injected intraperitoneally at 200 μg per mouse per injection, twice weekly. Tumor width (W) and length (L) were measured once a week using a caliper and tumor volume (V) was calculated according to the following formula: $V = 3.14 \times (W^2 \times L)/6$. Body weight was evaluated once per week. Mice were euthanized when tumor size reached 1,500 mm^3.

METHOD DETAILS

Analysis of patient tumor data

RNASeq data quantified in TPM scale for 1011 NSCLC tumor samples (LUAD, n = 513, LUSC, n = 498) and 1091 BRCA tumor samples was collected from the TCGA (https://xenabrowser.net/datapages/?dataset=tcga_RSEM_gene_tpm&host=https%3A%2F%2Ftoil.xenahubs.net&removeHub=https%3A%2F%2Fxena.treehouse.gi.ucsc.edu%3A443).

The data was then log2(TPM+1) transformed for further downstream analyses. Besides IFN-g signaling, we focused on 9 canonical growth factor signaling pathways to investigate if there exist additional cell extrinsic factors driving PD-L1 expression. Gene sets involved in these 9 different growth factor signaling pathways and the IFN signaling pathway were downloaded from the REACTOME database (https://reactome.org/). Using the log transformed expression values as input, pathway enrichment scores for each pathway were estimated in each sample using GSVA analysis tool (https://bioconductor.org/packages/release/bioc/html/GSVA.html). In order to assess the unique contributions of each pathway to PD-L1 expression, the pathway enrichment scores corresponding to each pathway were scaled and incorporated into the following multi-variate linear regression model:

$$PDL1\ expression \sim \sum_{i=1}^{10} \beta_i * P_i + \varepsilon$$

Here, P_i denotes the scaled pathway level enrichment score of one of the 10 pathways discussed above. ε denotes the intercept and the regression coefficients β denote partial correlations of PD-L1 expression with the estimated pathway activity. Note that we made available the scripts required to reproduce our results: https://github.com/ruppinlab/EGFR_NSCLC_ICB_analysis

Cell cycle analysis

Cell cycle distribution was determined using flow cytometry (FACSAria Fusion). Data were analyzed using Diva software v8.0.1 (BD Biosciences).

TEM and migration assays

For trans-endothelial migration (TEM) assays we used Corning's FluoroBlok multiwell insert plates with 8.0 μm pores. The apical side of the insert was coated with growth factor reduced basement membrane extract (10 μg/ml). Next, human vascular endothelial cells (HUVECs; 5 X 10^4 /well) were seeded on the coated inserts. CellTracker Green CMFDA-labeled PC9ER (WT) or PD-L1 KO cells (4 X 10^4) were overlaid 24 hours later. Labeled cells that migrated, invaded, and transendothelial migrated across the tissue barrier were photographed and quantified 22 hours later, using a bottom reading fluorescence plate reader. For migration and invasion assays, cells (4X10^4) were plated in the upper compartment of a 24-well chamber (Corning, Acton, MA) with an intervening nitrocellulose membrane (8 μm pore size).

Cell lysis, immunoblotting, and co-immunoprecipitation assays

Cell lysates were collected in a mild lysis buffer (50 mM HEPES, pH 7.5, 10% glycerol, 150 mM NaCl, 1% Triton X-100, 1 mM EDTA, 1 mM EGTA, 10 mM NaF and 30 mM β-glycerol phosphate). Proteins were immunoprecipitated from cell lysates using beads conjugated to an antibody. After 2 hours of incubation at 4°C, complexes were washed three times and bound proteins were eluted in 6X Laemmli buffer. Eluates were subjected to electrophoresis and immunoblotting. For immunoblotting, cleared cell lysates were resolved using electrophoresis, followed by electrophoretic transfer to a nitrocellulose membrane. Membranes were blocked with TBS-T (tris-buffered saline containing Tween-20) containing 1% low-fat milk, blotted overnight with a primary antibody, washed three times with TBS-T, incubated for 30 minutes with a secondary antibody linked to horseradish peroxidase, and washed once again with TBS-T. Immunoreactive bands were detected using the ECL reagent (Biorad).

RNA isolation and real-time PCR analysis

Total RNA was extracted using the RNeasy Mini Kit (QIAGEN, Hilden, Germany), according to the manufacturer's instructions. Total RNA quantity and quality were determined using the NanoDrop ND-1000 spectrophotometer (Thermo Fischer Scientific, Waltham, MA). Complementary DNA was synthesized using the High-Capacity Reverse Transcription kit (Applied Biosystems, Life Technologies, Carlsbad, CA, USA). Real-time qPCR analysis was performed with SYBR Green (Applied Biosystems) and specific primers on the StepOne Plus Real-Time PCR system (Applied Biosystems). qPCR signals (cT) were normalized to Actin.

Chemotaxis assays

PC9ER and PD-L1 knockout derivatives were cultured overnight on collagen. The left chamber was filled with media without serum and the right side was filled with media containing serum (1%) and EGF (30 ng/ml). Live imaging was performed for 16 hours and images were taken once every 15 minutes.

In vitro knockout of the gene encoding for PD-L1

The CRISPR system, along with Cas9, was used to create a double-stranded break next to the Protospacer Adjacent Motif (PAM) sequence. The selected target was 21bp long, including the PAM sequence in exon 2, which was filtered to minimize off-target cross-reactivity.

Proximity ligation assays (PLA)

PC9ER cells were grown on glass coverslips and fixed in PFA (4%) for 30 minutes, washed with saline and permeabilized for 10 minutes with saline containing Triton X-100 (0.4%). Cells were incubated for 60 minutes with primary antibodies specific to PD-L1 and PLC-G1, and this was followed by secondary antibodies, either Rabbit PLUS (DUO92002) or Mouse MINUS (DUO92002). The slides were processed using the Duolink *In Situ* Detection Kit (red) containing a tetramethylrhodamine-5-isothiocyanate (Sigma-Aldrich). Thereafter, cells were hybridized with phalloidin-FITC and DAPI, for counterstaining. Coverslips were washed and placed, cells face down, onto drops of an anti-fade reagent (from Dako). Samples were examined using a confocal microscope LSM 800 (Zeiss). Red dots and nuclei were counted and the number of positive stains per cell was calculated from at least 5 non-overlapping microscope fields.

Intracellular calcium measurements

Free cytosolic Ca^{2+} was examined using confocal microscopy or flow cytometry. Briefly, cells ($1X10^6$ cells) were washed in $PBS^{-/-}$ and incubated for 30 minutes with Calcium Orange (2 μM), washed and placed on the recording stage of the Pascal confocal microscope. For measurements using flow cytometry, Indo-1 acetoxymethyl ester (Abcam) was loaded into cells at a concentration of 10 μM (30 min at 37°C). Thereafter, cells were washed twice with $PBS^{-/-}$ before analysis. The 395/510 nm fluorescence ratio for the dye-loaded viable cells was determined using LSRII (Becton Dickinson Immunocytometry Systems). EGF induced changes in violet/blue ratio were determined by subtracting the mean fluorescence ratio of baseline Ca^{2+} from the mean fluorescence obtained after cell stimulation.

Lung metastasis assays

Cells ($1X10^6$) were injected in the tail vein of NSG mice (5-6 weeks old). Four weeks following injection, mice were injected intraperitoneally with d-luciferin dissolved in saline (0.05 mL of a 30 mg/ml solution, from Xenogen), and animals were anesthetized. Mice were sacrificed 5 minutes later and their lungs were excised prior to imaging that used IVIS Spectrum (Xenogen).

QUANTIFICATION AND STATISTICAL ANALYSIS

All data were analyzed using the Prism GraphPad software and statistical analyses were performed using one or two-way ANOVA with the Dunnett's or Tukey's test (*, $p < 0.05$; **, $p < 0.01$; ***. $p < 0.001$; ****, $p < 0.0001$). Flow cytometry analysis was performed on a BD FACSAria Fusion Instrument controlled by BD FACS Diva software v8.0.1 (BD Biosciences). Further analysis was performed using the FlowJo software v10.2 (Tree Star). Staining intensities were determined using ImageJ. Statistical details of experiments can be found in figure legends.

Cell Reports, Volume *35*

Supplemental information

PD-L1 recruits phospholipase C and enhances tumorigenicity of lung tumors harboring mutant forms of EGFR

Soma Ghosh, Nishanth Belugali Nataraj, Ashish Noronha, Sushant Patkar, Arunachalam Sekar, Saptaparna Mukherjee, Sabina Winograd-Katz, Lior Kramarski, Aakanksha Verma, Moshit Lindzen, Diana Drago Garcia, Joseph Green, Galit Eisenberg, Hava Gil-Henn, Arkaprabha Basu, Yan Lender, Shimon Weiss, Moshe Oren, Michal Lotem, Benjamin Geiger, Eytan Ruppin, and Yosef Yarden

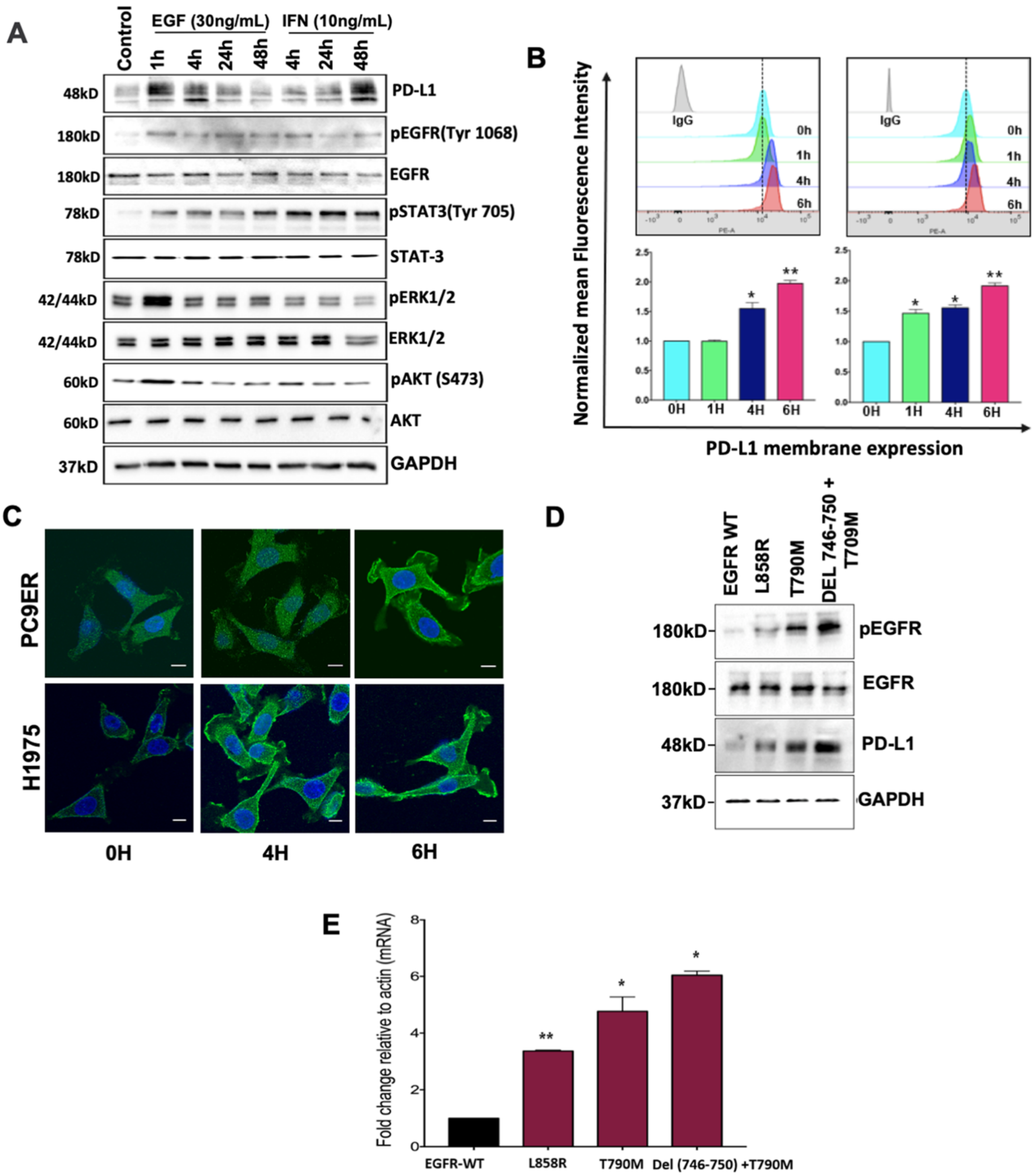

Figure S1: Delayed and early induction of PD-L1 by IFN-g and EGF (respectively), related to Figure 1. (**A**) Serum-starved PC9ER cells were treated for the indicated time intervals with EGF or IFN-

g. Cleared extracts were immunoblotted for the indicated proteins. (**B**) PC9ER (left) and H1975 (right) cells were stimulated with EGF (30 ng/ml) and later analyzed for PD-L1's surface expression using flow cytometry. Normalized surface levels from 3 experiments are shown. (**C**) PC9ER and H1975 cells ($0.5X10^6$) were seeded on coverslips and allowed to grow for 24 hours. Cells were serum starved for 16 hours, treated with EGF (30 ng/ml), as indicated, and then fixed in formaldehyde (4%). Thereafter, cells were incubated overnight with an anti-PD-L1 primary antibody (green), followed by a secondary, FITC-conjugated antibody. DAPI staining (blue) indicates locations of nuclei. Images were captured using a confocal microscope (63X magnification). Bars, 20 μm. Images are representative of two independent experiments. (**D**) NL20 lung epithelial cells were stably transfected with a vector (pLex307) encoding the indicated mutant forms of EGFR (L858R, T790M or the double mutant del (746-750) plus T790M). Cleared whole cell extracts were immunoblotted for the indicated proteins. GAPDH was used as loading control. Data are representative of two independent experiments. (**E**) The indicated cell lines were analyzed using qPCR for mRNA levels corresponding to PD-L1.

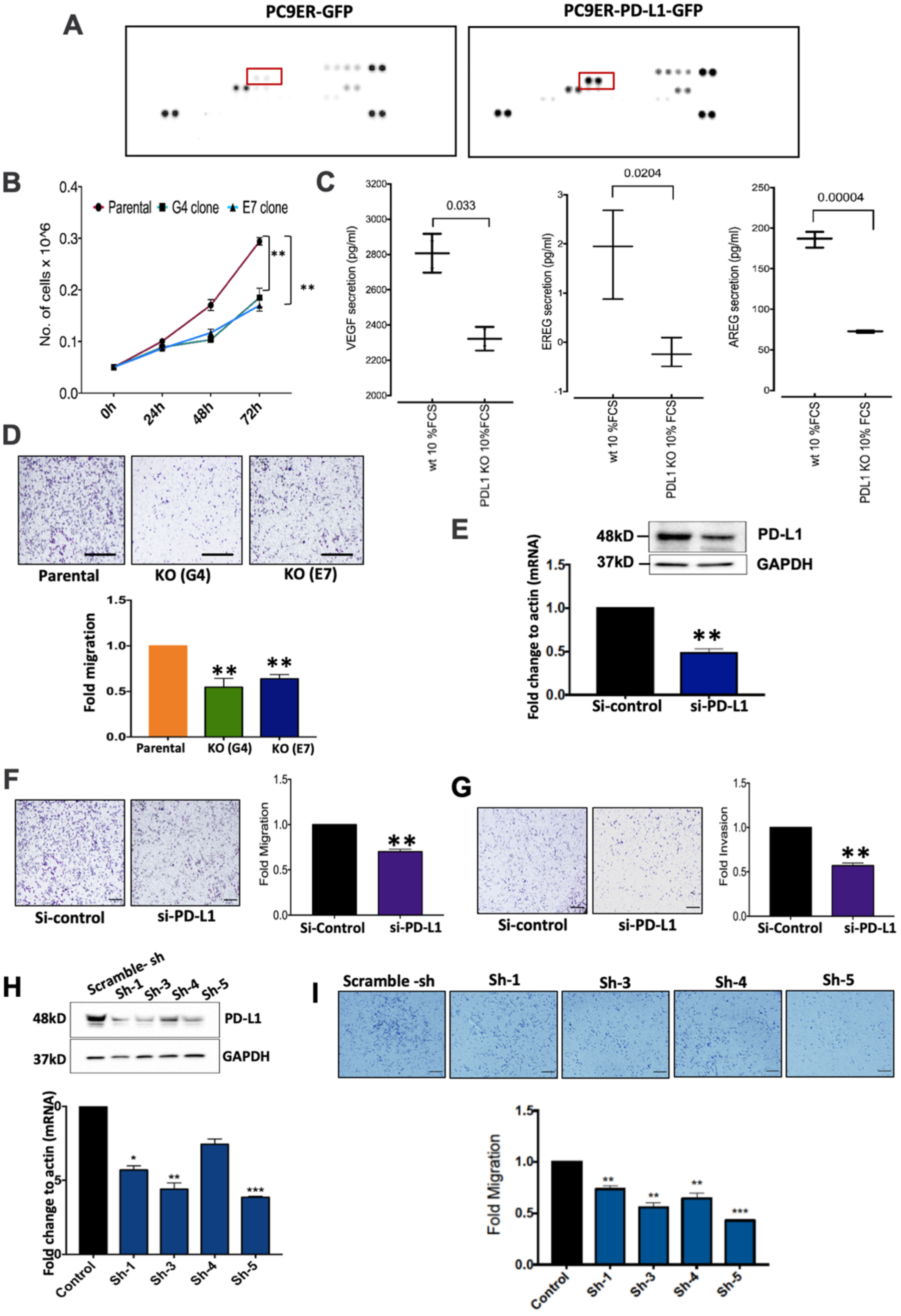

Figure S2: PD-L1 regulates proliferation, migration and invasion of NSCLC cells, as well as the secretome of lung cancer cells, related to Figures 3 and 4. (**A**) Serum-containing media were

conditioned over a period of 72 hours by control PC9ER-GFP cells or cells transiently overexpressing a PD-L1 protein fused to GFP. Cleared media were incubated with cytokine arrays (Proteome Profiler Human Cytokine Array, ARY005B; from R&D Systems). Each array was pre-spotted with 36 different antibodies specific to human cytokines (in duplicates). The arrays were processed according to the manufacturer's instructions. Duplicate spots corresponding to IL-18 are highlighted. (**B**) Parental PC9ER cells (PD-L1-WT; 8X10^4) and two derivatives PD-L1-KO cells were seeded onto 6-well plates for assessing cell proliferation. Cells were treated with trypsin and counted at the indicated time intervals. Results are presented as means ± S.D. of triplicates. The experiment was repeated thrice. (**C**) Serum-containing media were conditioned over a period of 72 hours by control PC9ER cells or by PD-L1 KO cells. ELISA kits (DuoSet, from R&D Systems), along with internal references, were used to determine concentrations of VEGF, epiregulin and amphiregulin. (**D**) Parental PC9ER cells and the PD-L1-KO clones were seeded in the upper compartment of Transwell migration chambers. Following 18 hours of incubation, four microscope fields were randomly selected for signal quantification. (**E**) PC9ER cells were transfected with siRNA oligonucleotides specific to PD-L1, or with control oligonucleotides (5 nM). Forty-eight hours later, cells were harvested in duplicates for immunoblotting and qPCR analysis. (**F** and **G**) PC9ER cells were treated with siRNAs as in E and 24 hours later they were seeded in the upper compartment of Transwell migration chambers (F) or in Matrigel-coated invasion chambers (G). Thereafter, complete medium was added into the lower compartments and cells were incubated for 18 hours at 37°C. Paraformaldehyde was used to fix cells that reached the lower side of the intervening filter, and crystal violet was used to stain the cells. Four microscope fields were randomly selected for signal quantification. (**H**) PC9ER cells were infected with lentivirus-based shRNAs, which were used to stably knockdown expression of PD-L1. Four different shRNA plasmids were used for transfection into PC9ER cells and four shPD-L1 stable clones were selected. Cells were harvested and extracts were used for either immunoblotting for PD-L1 expression, relative to shCTRL cells (upper panel), or for qPCR tests that assayed the levels of PD-L1's mRNA relative to GAPDH transcripts (lower panel). (**I**) Transwell migration assays were performed with shCTRL-transfected PC9ER cells, along with four different shPD-L1 clones. Bar graphs are presented as means + S.D. of triplicates.

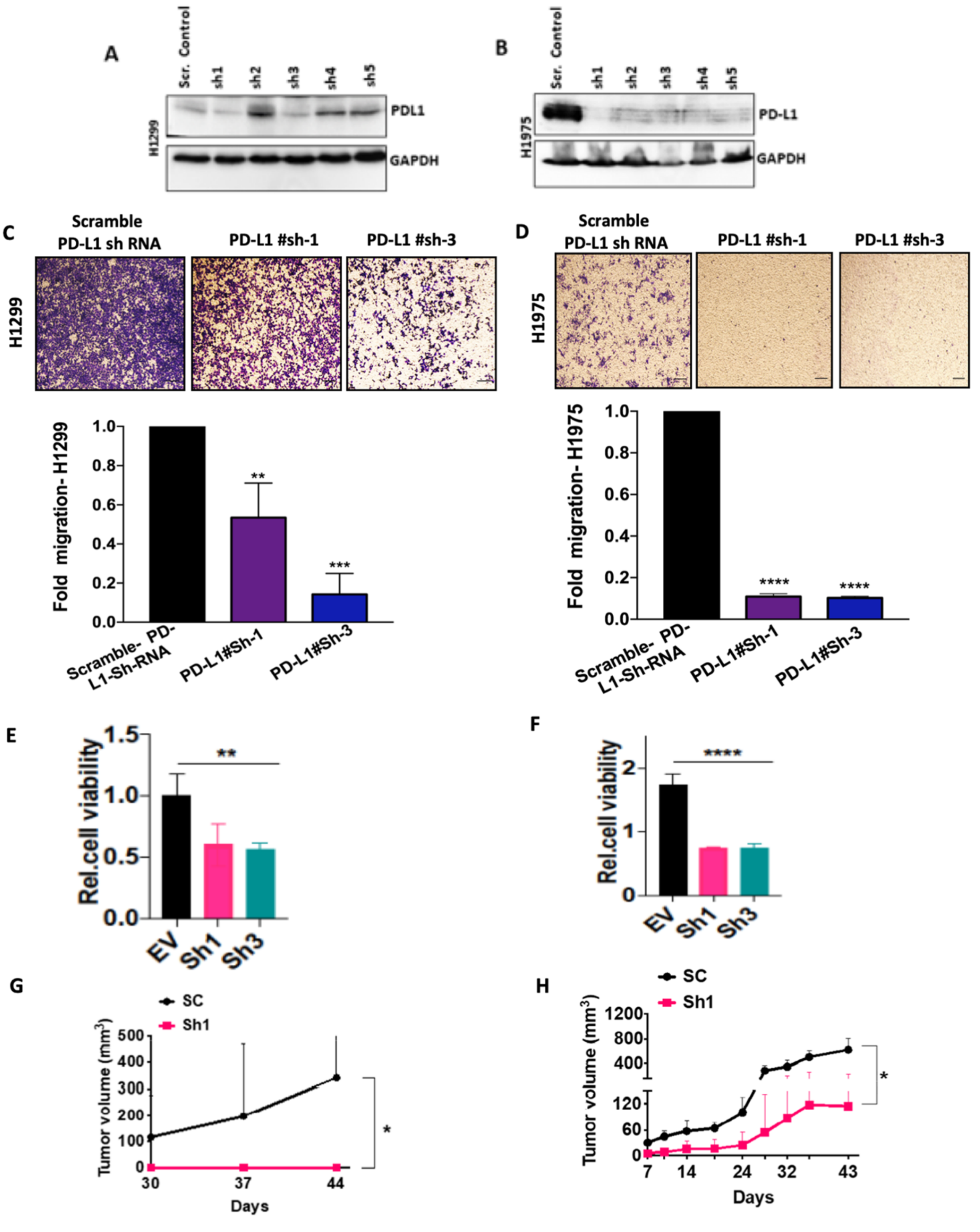

Figure S3: Reduced abundance of PD-L1 results in slower rates of migration, proliferation and tumorigenesis of cells expressing WT-EGFR or the L858R mutant form, related to Figure 4. (**A** and

B) H1299 (WT-EGFR; A) and H1975 (L858R-EGFR; B) cells were infected with lentivirus-based shRNAs, to stably knockdown expression of PD-L1. Five different shRNAs were introduced into each cell line. Cells were harvested and extracts were used for PD-L1 immunoblotting, relative to the Scrambled Control shRNA (SC). GAPDH served as the loading control. (**C** and **D**) Two independent shPD-L1 stable clones (Sh#1 and Sh#3) were selected and Transwell migration assays were performed in H1299 (left panel) and H1975 (right panel), relative to cells treated with the Scrambled Control shRNA (SC). Bar graphs show means + S.D. of triplicates. (**E** and **F**) MTT assays were performed to measure proliferation of H1299 (E) and H1975 cells (F) in which PD-L1 was stably knocked-down. Data presented are means + S.D. of triplicates. (**G** and **H**) shPD-L1 derivatives of H1299 (G) and H1975 cells (H), along with cells treated with Scrambled Control shRNA (SC), were implanted subcutaneously in athymic mice. Shown are averages + S.D. of tumor volumes.

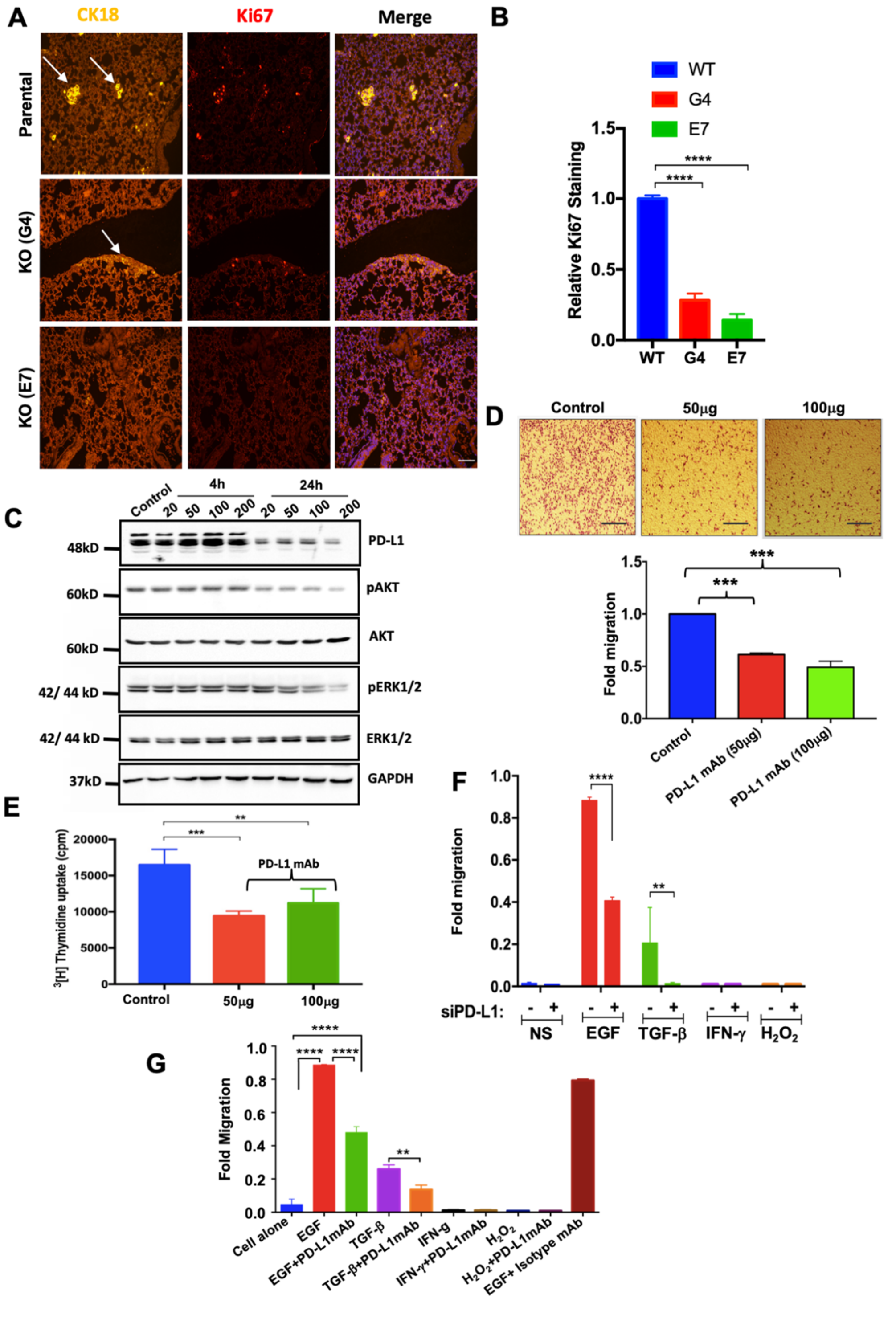

Figure S4: Genetic depletion of PD-L1 retards metastatic colonization of murine lungs, while antibody-induced depletion inhibits growth factor mediated cell proliferation and migration, related to Figure 4. (**A**) Parental PC9ER-LUC (WT), PDL1-KO-G4-LUC and PD-L1-KO-E7-LUC cells

(1X10^6 cells) were injected into the tail vein of male NSG mice. Ten days post implantation, mice were injected intraperitoneally with 0.05 ml D-Luciferin solution (15 mg/ml, in saline), 5 minutes prior to luminescence measurements. Lungs were excised, fixed in formalin, and paraffin embedded. Sections of 0.2 mm were deparaffinized and immunostained for Ki67. Likewise, we probed thin slices with an antibody specific to the human form of cytokeratin 18. White arrows mark CK18 positive cells. (**B**) Proliferation was quantified by counting Ki67-positive cells in five representative microscope fields per lung (from A). Three lungs were surveyed per group. Scale bars, 0.1 mm. (**C**) PC9ER cells were incubated in the presence of increasing concentrations of durvalumab (20-200 µg/ml). Cells were harvested at the indicated time points. Cleared cell extracts were resolved and immunoblotted as indicated. (**D**) PC9ER cells (4X10^5) were pre-treated for 24 hours with durvalumab (0.1 mg/ml or 0.05 mg/ml) and later subjected to a cell migration assay (18 hours). Cell images and average signals are shown. (**E**) PC9ER cells were plated onto 24-well plates at 1X10^4 cells/well and treated for 48h with durvalumab (0.1 mg/ml or 0.05 mg/ml). Thereafter, the media were refreshed and 3H-thymidine (1 µCi) was added. The incubation was terminated 48 hours later. Shown are averages + S.D. of quadruplicates. (**F**) MCF10A cells were transfected with siPD-L1 or control oligonucleotides, and 36 hours later cells (4X10^4) were plated in the upper compartment of 24-well Transwell chambers, in the absence of serum. Thereafter, the serum-free medium in the lower compartment of the chamber was supplemented with growth factors (EGF, 30 ng/ml; TGF-b1, 30 ng/ml; IFN-g, 10 ng/ml) or H2O2, 10 µM). Cells were allowed to migrate for 22 hours at 37°C through the intervening nitrocellulose membrane (8-µm pore size). The filter was later removed, and attached cells were fixed for 15 minutes in saline containing paraformaldehyde (4%). Staining with crystal violet followed this step. Cells growing on the upper side of the filter were removed, while cells located on the bottom side were photographed and optical density was measured at 595 nm. Representative microscope fields were taken, and signals were quantified (means + SEM). (**G**) MCF10A cells (4X10^5) were pre-treated for 24 hours with either durvalumab (0.05 mg/ml) or an isotype-matched antibody. Thereafter, the cells were suspended in serum free medium containing the respective antibody and seeded in the upper compartment of Transwell chambers. In parallel, the lower compartment was supplemented with the indicated growth factors/agents (EGF, 30 ng/ml; TGF-b, 30 ng/ml; IFN-g, 10 ng/ml or H2O2, 10 µM) or medium alone. Cells were allowed to migrate for 22 hours. Signal quantification (average + SEM) and representative fields are presented.

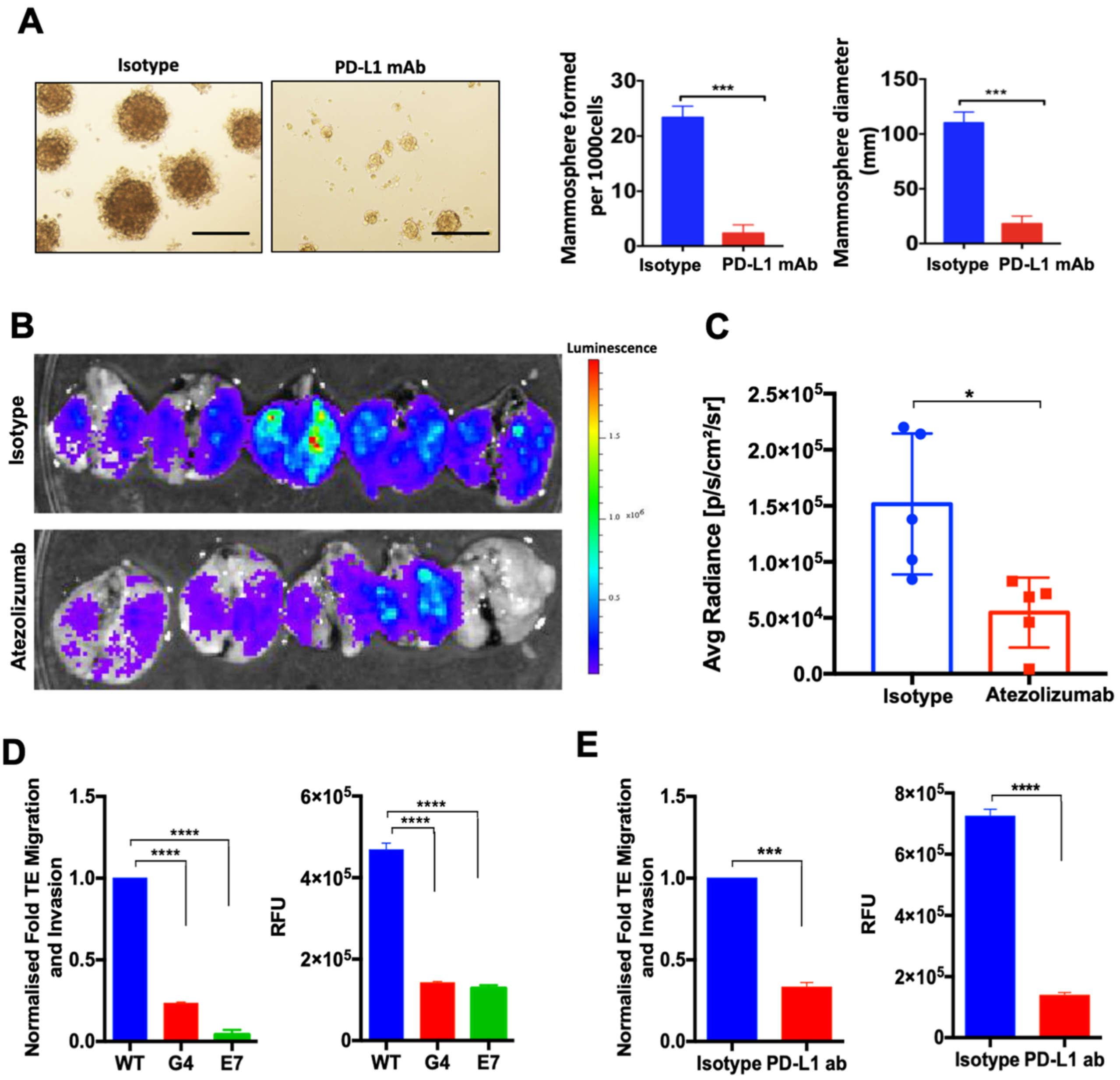

Figure S5: Anti-PD-L1 antibodies inhibit sphere formation by mammary cells, as well as transendothelial migration and metastasis of NSCLC cells, related to Figure 4. (**A**) MCF10A cells (1,000 cells per well) grown in mammosphere medium were seeded in ultra-low attachment plates in the presence of an anti-PD-L1 antibody (durvalumab; 0.05 mg/ml) or an isotype matched antibody. Photographs were captured two weeks later and spheres larger than 60 μm in size were measured and counted. (**B**) PC9ER cells ($1X10^6$) were injected into the tail vein of NSG mice. Forty-eight hours later, mice were randomized: one group (n=5) was intraperitoneally injected

with atezolizumab (0.2 mg per mouse) and the other received a control antibody (isotype matched). Injections were repeated on days 3, 6, 9, 12, 14, 16 and 18. On day 20, mice were treated with luciferin and 5 minutes later their lungs were resected. Lungs were visualized using the IVIS Spectrum In Vivo Imaging System (Perkin Elmer). (**C**) Quantification of the radiance signals from B. (**D**) Migration was assayed in Corning FluoroBlok multiwell insert plates with 8.0 µm pores. Prior to seeding in the insert, the apical side of the insert was coated with a basement membrane preparation (10 µg/ml, growth factor reduced). Human vascular endothelial cells (HUVECs; $5X10^4$ /well) were added into the coated inserts. EGF (30 ng/ml) was added in the base chamber as a chemo-attractant. After 24 hours, $4X10^4$ CellTracker Green CMFDA-labeled PC9ER or PD-L1 KO cells (G4 and E7) were added. After 22 additional hours, transendothelial migrated and invaded cells were imaged, and fluorescence intensity was quantified. (**E**) The protocol used in D was repeated except that cells were pretreated for 48 hours with an anti-PD-L1 antibody (durvalumab; 50µg/ml). Thereafter, the cells were added into the inserts in the presence of the antibody.

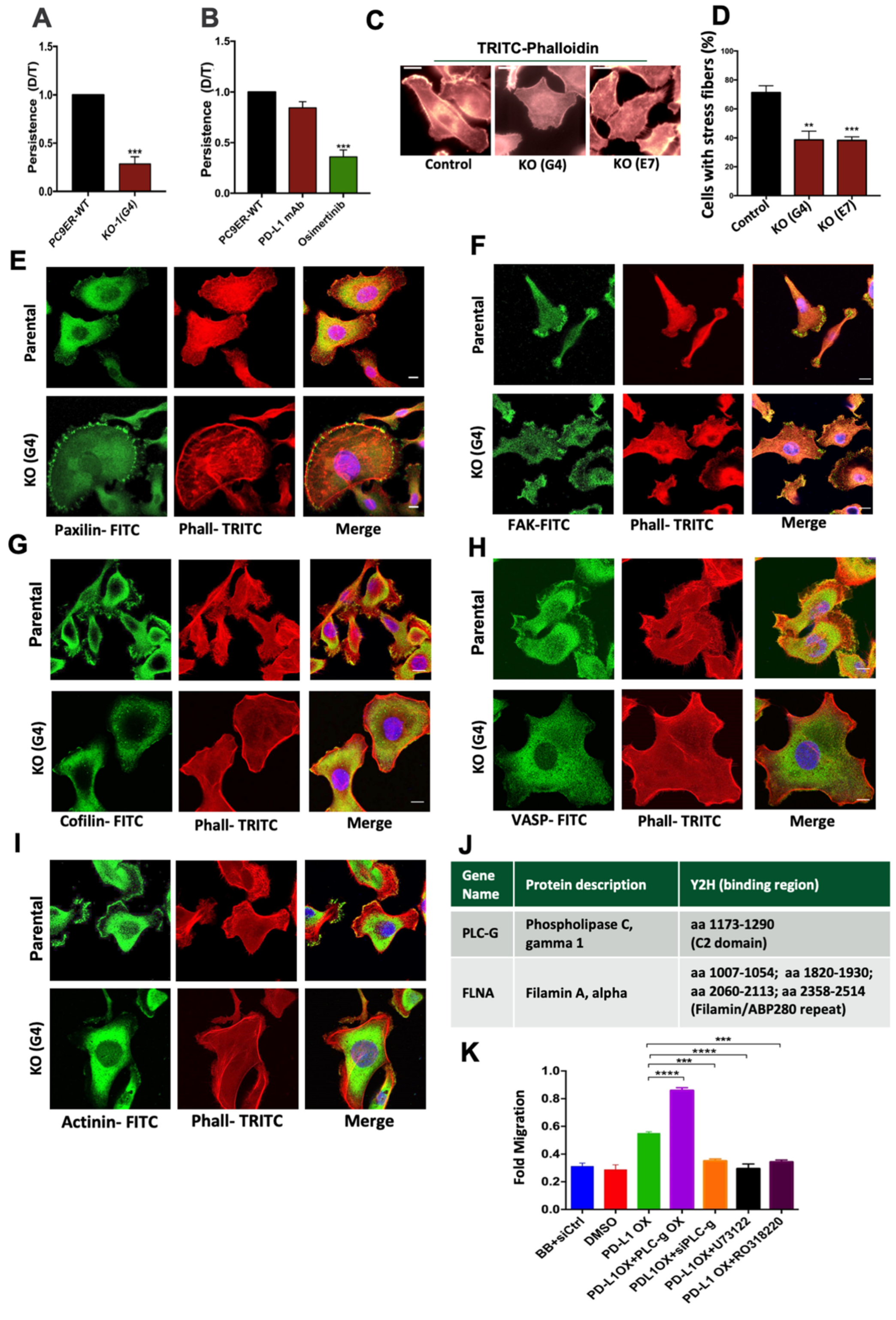

Figure S6: PD-L1 regulates migration persistence and lamellipodia formation, likely by recruiting PLC-g1 and FLNA, related to Figure 5. (**A** and **B**) Chemotaxis assays of PC9ER and PD-L1-KO

cells were performed as described in Figs. 5A. Data from the respective rose plots were processed and scores of migration persistence were calculated by following migration of twenty cells per assay. (**C** and **D**) Cells (0.5X10^6) were seeded on coverslips and allowed to grow for 48 hours in media containing TGF-b (5 ng/ml). Samples were fixed with paraformaldehyde (4%), permeabilized with Triton-X (0.1%) and stained with Actin-stain 488 phalloidin (from Cytoskeleton Inc.). After mounting, the cells were imaged using a Nikon Eclipse Ti widefield microscope. Images were taken at 63X magnification and quantified in at least 6 non-overlapping fields of four independent experiments. (**E-I**) Control PC9ER cells (0.5X10^6) and cells lacking PD-L1 were seeded on coverslips and allowed to grow for 24 hours. Thereafter, cells were fixed and incubated overnight with antibodies specific to paxilin, FAK, cofilin, VASP and actinin. This was followed by a secondary, FITC-conjugated antibody. DAPI was used to visualize nuclei. Images were taken using confocal microscopy (63X magnification). Bars, 10 μM. (**J**) Yeast two-hybrid screens were performed using the portion of the human *PD-L1* cDNA that encodes the full-length cytoplasmic domain. This segment was cloned into a bait construct as an N-terminal fusion to LexA. The construct was used to screen a human lung cancer cDNA library and positive clones were selected. The respective prey fragments were amplified and sequenced. The resulting sequences were used to identify the corresponding interacting proteins. Listed are the two strongest interactors and their putative binding sites. (**K**) PC9ER cells were transfected with plasmids encoding PLC-g1 and PD-L1. Twenty-four hours later, some plates were transfected with siPLCg1, or siCtrl, and following 24 hours cells (4X10^4) were plated in the upper compartment of a 24-well Transwell tray. We used specific inhibitors of PLC-g1 (U73122) and PKC-a (RO 318220) at 2 μM throughout the assay, as indicated. The control group received DMSO alone. Cells were allowed to migrate for 18 hours through the intervening nitrocellulose filter (8-μm pore size). The filter was later removed and attached cells were fixed for 15 minutes in saline containing paraformaldehyde (4%). Cells located on the bottom side were photographed and quantified in triplicates (+ SEM). Bars, 500 μm

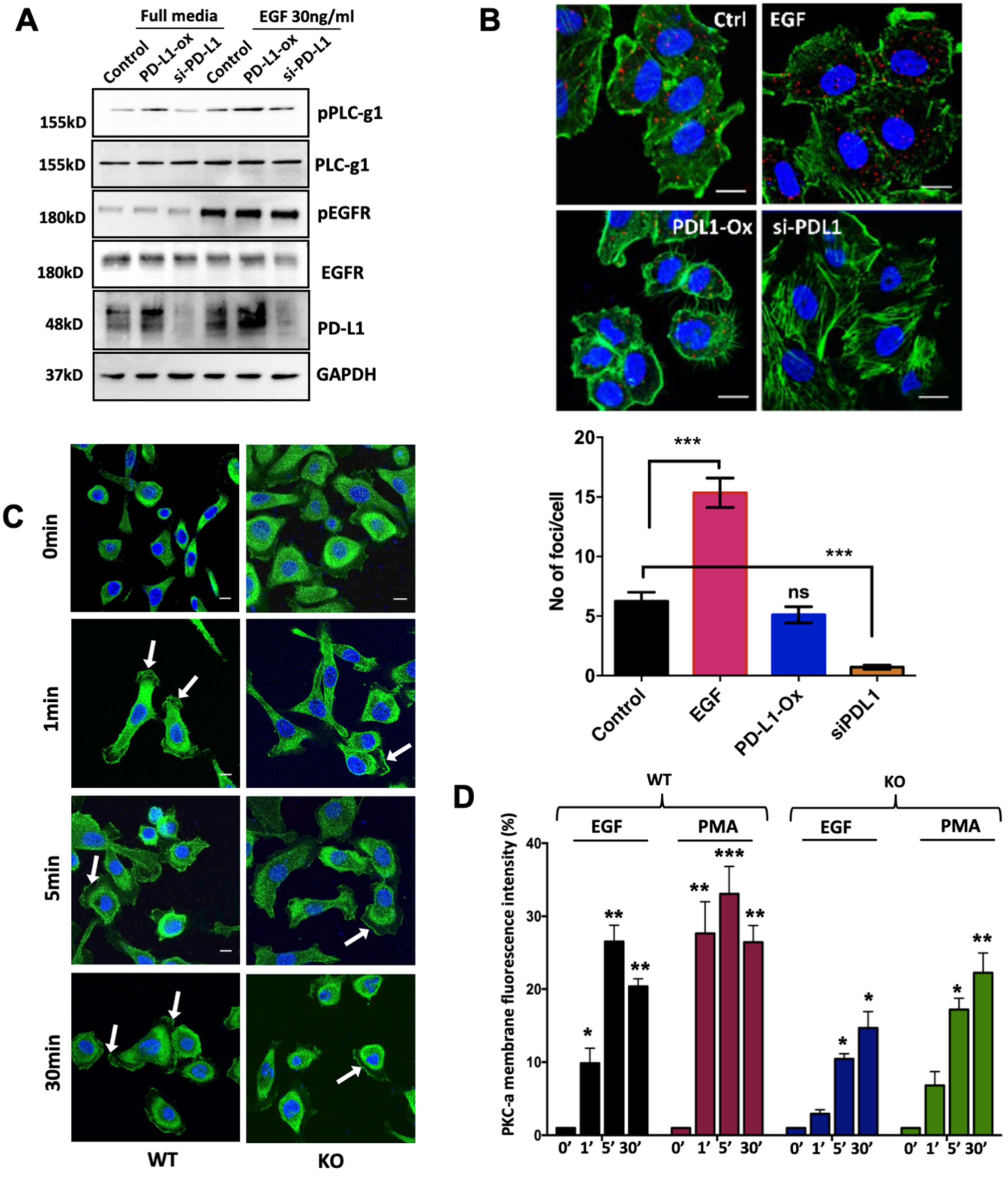

Figure S7: Cells depleted of PD-L1 are unable to activate PLC-g1 and its downstream signaling, related to Figure 5. (**A**) PC9ER cells were pre-transfected with siPD-L1 or with a plasmid encoding

PD-L1. Following 48 hours from transfection, cells were incubated with EGF and 30 minutes later their extracts were analyzed using the indicated antibodies. (**B**) PC9ER cells were pre-transfected with siPD-L1 or a plasmid encoding PD-L1, and 48 hours after transfection they underwent fixation. In parallel, parental PC9ER cells were untreated or treated for 30 minutes with EGF (30 ng/ml), prior to fixation. Similarly, cells lacking PD-L1 expression were fixed. Thereafter, all fixed cells were probed with antibodies recognizing PD-L1 and PLC-g1, and later processed for proximity ligation assays (PLA) that used a tetramethylrhodamine-5-isothiocyanate (TRITC) as a probe (red). Counterstaining used DAPI (blue) and phalloidin-FITC (green). The number of dots per cell were quantified using Image J (below panel). Scale bar, 10 µm (ns, not significant). (**C**) Parental PC9ER cells ($0.5X10^6$) and cells devoid of PD-L1 (KO) were seeded on coverslips. Cells were serum starved for 16 hours, and then treated with PMA (30 nM) for the indicated time intervals. Cells were washed in saline containing Tween 20, fixed and permeabilized. Thereafter, cells were fixed and incubated with a PKC-a specific antibody, followed by a secondary, FITC-conjugated antibody. Images were taken using confocal microscopy (63X magnification). Arrows mark PKC-a molecules recruited to the plasma membrane. Bars, 10 µm. (**D**) Cells were treated with EGF (30 ng/ml) or PMA (30 nM), and PKC-a recruitment to the plasma membrane was determined using immunofluorescence.
